# Establishment of Elevated Serum Levels of IL-10, IL-8 and TNF-β as Potential Peripheral Blood Biomarkers in Tubercular Lymphadenitis: A Prospective Observational Cohort Study

**DOI:** 10.1371/journal.pone.0145576

**Published:** 2016-01-19

**Authors:** Abhimanyu, Mridula Bose, Mandira Varma-Basil, Ashima Jain, Tavpritesh Sethi, Pradeep Kumar Tiwari, Anurag Agrawal, Jayant Nagesh Banavaliker, Kumar Tapas Bhowmick

**Affiliations:** 1 Department of Microbiology, Vallabhbhai Patel Chest institute, University of Delhi, New Delhi, India; 2 CSIR-Institute of Genomics and integrative Biology, New Delhi, India; 3 RajanBabu Institute of Pulmonary Medicine and Tuberculosis, Kingsway Camp, New Delhi, India; 4 Department of Radiotherapy, Vardhman Mahavir Medical College and Safdarjung Hospital, Ansari Nagar west, New Delhi, India; The Ohio State University, UNITED STATES

## Abstract

**Background:**

Tubercular lymphadenitis (TL) is the most common form of extra-pulmonary tuberculosis (TB) consisting about 15–20% of all TB cases. The currently available diagnostic modalities for (TL), are invasive and involve a high index of suspicion, having limited accuracy. We hypothesized that TL would have a distinct cytokine signature that would distinguish it from pulmonary TB (PTB), peripheral tubercular lymphadenopathy (LNTB), healthy controls (HC), other lymphadenopathies (LAP) and cancerous LAP. To assess this twelve cytokines (Tumor Necrosis Factor (TNF)—α, Interferon (IFN) -γ, Interleukin (IL)-2, IL-12, IL-18, IL-1β, IL-10, IL-6, IL-4, IL-1Receptor antagonist (IL-1Ra), IL-8 and TNF-β, which have a role in pathogenesis of tuberculosis, were tested as potential peripheral blood biomarkers to aid the diagnosis of TL when routine investigations prove to be of limited value.

**Methods and Findings:**

A prospective observational cohort study carried out during 2010–2013. This was a multi-center study with three participating hospitals in Delhi, India where through random sampling cohorts were established. The subjects were above 15 years of age, HIV-negative with no predisposing ailments to TB (n = 338). The discovery cohort (n = 218) had LNTB (n = 50), PTB (n = 84) and HC (n = 84). The independent validation cohort (n = 120) composed of patients with cancerous LAP (n = 35), other LAP (n = 20) as well as with independent PTB (n = 30), LNTB (n = 15) and HC (n = 20). Eight out of twelve cytokines achieved statistical relevance upon evaluation by pairwise and ROC analysis. Further, variable selection using random forest backward elimination revealed six serum biosignatures including IL-12, IL-4, IL-6, IL-10, IL-8 and TNF-β as optimal for classifying the LNTB status of an individual. For the sake of clinical applicability we further selected a three analyte panel (IL-8, IL-10 and TNF-β) which was subjected to multinomial modeling in the independent validation cohort which was randomised into training and test cohorts, achieving an overwhelming 95.9% overall classifying accuracy for correctly classifying LNTB cases with a minimal (7%) misclassification error rate in the test cohort.

**Conclusions:**

In our study, a three analyte serum biosignatures and probability equations were established which can guide the physician in their clinical decision making and step wise management of LNTB patients. This set of biomarkers has the potential to be a valuable adjunct to the diagnosis of TL in cases where AFB positivity and granulomatous findings elude the clinician.

## Introduction

Cytokine homeostasis in tuberculosis is an area of intense research. At the same time the understanding of immunological responses in tuberculosis still remains incomplete. Although all the research focus has been directed to pulmonary tuberculosis (PTB), a pertinent world health problem, extra-pulmonary TB (EP-TB) has its fair share of tuberculosis burden [1]. The Indian prevalence of EP-TB coincides with the global prevalence and constitute about 15–20% of all cases of TB in immunocompetent cases and 50% in cases infected with Human Immunodeficiency Virus (HIV) [2]. The most common form of EP-TB is tubercular lymphadenitis (LNTB) with 50% of the cases involving the peripheral lymph nodes [3–4]. The diagnosis of LNTB involves a high index of suspicion and invasive fine needle aspiration (FNA). Often biopsy becomes necessary because the symptoms and disease presentation are misleading [5]. While newer nucleic acid based diagnostic tests have been reported for EP-TB including LNTB highly variable sensitivity and specificity leaves them to be improved further. Moreover, the FNA samples are usually paucibacillary decreasing the sensitivity of the diagnostic test further [6]. It is therefore pertinent to search for some dependable biomarkers for LNTB that would be of diagnostic value to replace the conventional techniques with a less invasive one or as an adjunct to strengthen the diagnosis.

Search for immunological biomarkers in tuberculosis has been largely guided by the Th1/Th2 paradigm. But of late, the disease has been recognized as complex continuous spectrum of overlapping immunological responses [7–8]. The aim of the present study was to assess a wide spectrum of cytokines to explore possible biosignatures for LNTB to discern such cases from PTB, HC and other LAP. We propose that dependable biosignature in conjunction with the available techniques of FNA, smear microscopy and culture test could aid in definitive diagnosis of the disease. Multi analyte panels of biomarkers offer clear statistical advantages over individual biomarkers for diagnostic and prognostic use across a variety of diseases [9]. The “combinatorial” approach used in the present study has been hailed as the “paradigm shift” in the tuberculosis biomarker discovery [10].

The available literature dealing with probable immunological biomarkers focus solely on pulmonary tuberculosis [11]. Host serum cytokine responses in EP-TB have been reported in a few studies [12–15] but these studies have considered a limited number of samples from different manifestations of EP-TB. More importantly, reports that have investigated PTB and LNTB cases from the same feeder population for a comparative analysis are not available. Even a recent study measuring the serum levels of IFN-γ, chemokine ligand 9, mannose-binding lectin (MBL), and tumor marker Ca-125 found no difference in profile between PTB and LNTB [16].

The present study to the best of our knowledge brings out for the first time a comparative profile of serum cytokine responses in LNTB and PTB in HIV negative individuals. Twelve pro and anti-inflammatory cytokines indicated to have a role in tuberculosis infection and control, namely TNF-α, Interferon (IFN)-γ, IL-2, IL-18, IL-1β, IL-10, IL-4, IL-1Ra, IL-8 [17] and TNF-β [18] have been taken up for analysis here. For clinical applicability, interpretability and the validity of the panel, extensive modeling approaches using statistics and machine learning models were applied. The results present a clear insight into the profile of serum cytokine response in LNTB and contribute a number of markers of immunological value to diagnose lymph node tuberculosis. We demonstrate that elevated IL-10, IL-8 and TNF-β levels in the serum of lymph node tuberculosis patients may be a robust indicator of the disease. This information could be a valuable adjunct to the conventional modalities currently in use in the diagnosis of LNTB. The results also emphasizes the need to explore beyond the classical Th1/Th2 paradigm in tuberculosis biomarker research. The proposed serum test could be used as initial screen to assess LNTB risk and based on it a smaller number of patients can be subject to FNA for further screening. Alternatively, this serum test is also capable of distinguishing the LNTB with other LAP including malignancies thereby eliminating the need for repeated FNA when there are no conclusive result of the initial FNA.

## Methods

### Study Population and Laboratory Methods

**Fig 1:**
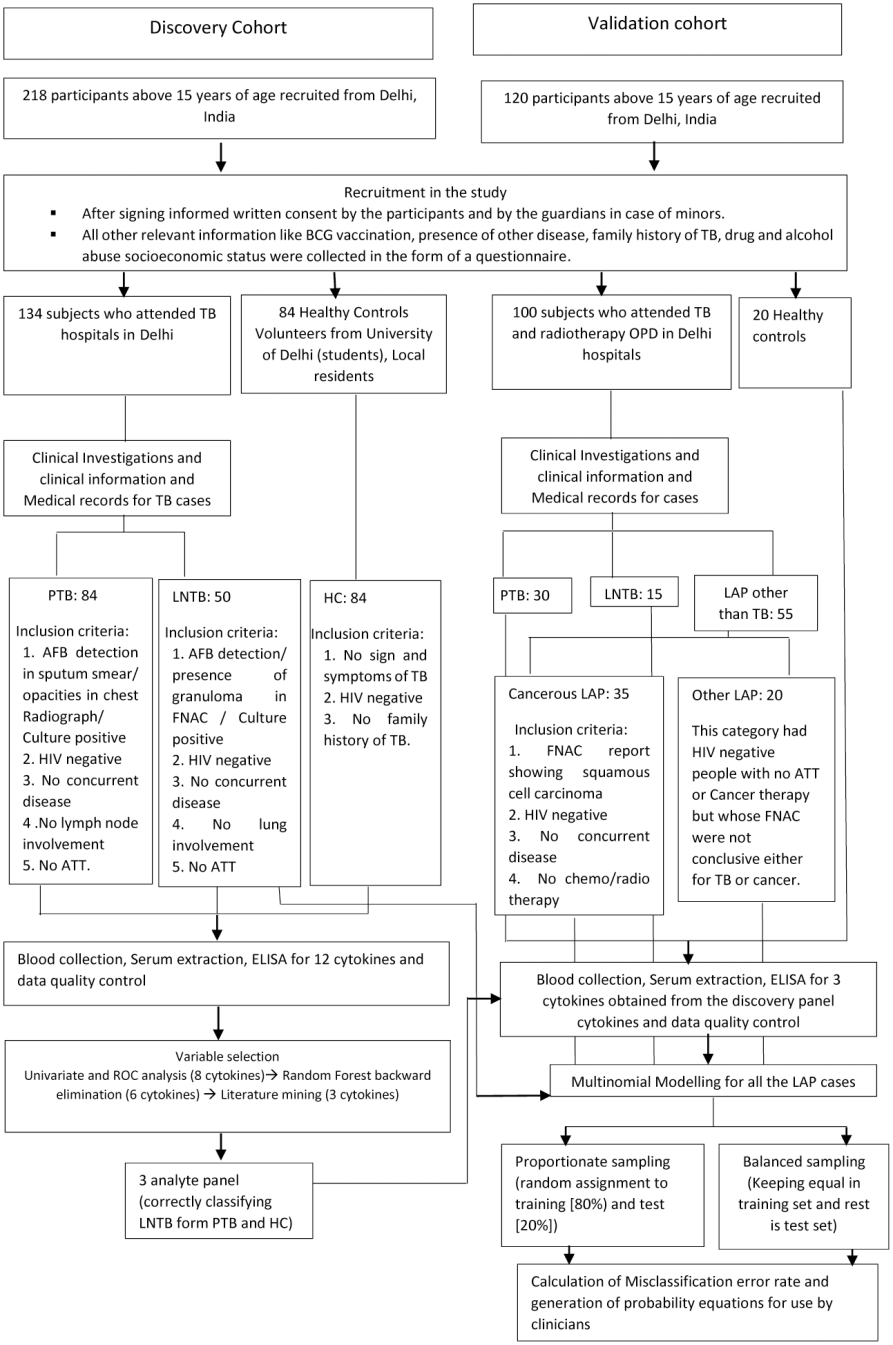
Overall Study Design including the numbers of subjects included in the Discovery and Validation Cohorts. The indicated study design also enlists the inclusion criteria for different classes of subjects in the discovery and validation cohorts. The cytokines selected from the discovery cohort were tested in the validation cohort. Among the LAP subjects in the validation cohort, LNTB subjects from discovery cohort were also included for multinomial modelling to check for their accurate classification by the model. Two approaches namely the balanced and proportionate sampling were used. The model was trained using the cytokine levels of the subjects from training set and then applied to a test set to determine their discriminating ability. The LNTB patients were attempted for a follow-up after the completion of their ATT therapy but only eight subjects could be enrolled.

The patient recruitment criteria and the description of the discovery and validation cohorts is shown in Fig 1.

### Initial discovery Cohort

Eighty-four freshly diagnosed sputum positive cases of pulmonary tuberculosis (PTB) and 50 lymph node tuberculosis cases above 15 years of age were enrolled in the study before start of any anti-tubercular therapy and within 15 days of complaint from the Rajan Babu Institute of Pulmonary Medicine and Tuberculosis (RBIPMT), Kingsway camp, New Delhi, India and the Chest clinic, Lok Nayak hospital, Delhi gate, New Delhi, India between January 2010 and January 2012. The pulmonary tuberculosis cases had undergone clinical and radiological (chest X-ray) diagnosis and had been confirmed by sputum microscopy for Mycobacteria at the respective hospitals as per the guidelines of Revised National TB Control Program (RNCTP), Ministry of Health and family Welfare, The Government of India. Clinical data were obtained from patients’ medical records. Fine needle aspiration cytology (FNAC) for enlarged lymph nodes was undertaken at respective hospitals. The sputum and FNAC samples were brought and direct smear microscopy was repeated and culture was performed at Department of Microbiology, V P Chest institute. The culture positive isolates were biochemically confirmed as *Mycobacterium tuberculosis*. All cases had access to free anti-tubercular drugs under DOTS (Directly Observed Treatment, short course) regimen of the Government of India. Cases with any immunosuppressive presentation such as diabetes mellitus or HIV co-infection which are considered to be risk factors for tuberculosis development and cases suspected of extra-pulmonary involvement along with pulmonary TB were excluded from the study. The control (HC) group consisted of 84 randomly chosen healthy students and laboratory personnel from the various departments of the University of Delhi, enrolled in the study following informed consent. They had no signs, symptoms or history of mycobacterial infection. HC were not subjected to tuberculin skin testing or IGRAs as the study was not focused on studying PTB, where presence of a latent infection would confound the results. Also most people in the HC were vaccinated with BCG so tuberculin would have been inconclusive.

Enrolled patients were briefed about the study and a signed informed consent was obtained from the patient or his /her guardians before sample collection. The study was approved by the ethical committee of the Vallabhbhai Patel Chest Institute, University of Delhi, India in accordance with the Indian Council of Medical Research (ICMR) guidelines. Baseline demographics, clinical and pathological characteristics of the study groups are mentioned in Table 1.

**Table 1:** Showing Demographical and Clinical Characteristics of the Study Groups.

Study Subject Classification	Demographics and Clinical Information	Discovery Cohort	Validation Cohort		
	**Demographics**				
		**LNTB**	**LNTB**	**Cancerous LAP**	**Other LAP**
	n	50	15	35	15
	Age in years(mean ± SEM)	25 ±1.7	26 ± 2	52 ± 11	35± 10
	Gender male (%): female (%)	21(42):29 (58)	8 (53):7 (46)	27 (76): 8(24)	8 (53):7 (46)
	**Primary diagnosis**				
	Location of lymph node (n)	C/+ A = (25) /+ (2); A = (6); SC/ + C = (5) /+ (3); SM = (5); I = (1); Su = (1); P/RA = (1/1)	C = (14); SS = (1)	C = (30); SM = (2); SC = (3)	C = (15)
	Size of lymph node (cm)	L (1.2); S (0.2)	L (1.3); S (0.3)	L (8); S (1.5)	L (4); S (1.3)
**Lymphadenopathy**	Tenderness	T (35); N (15)	T (10); N (5)	T (21); N (14)	T (11); N (4)
	Matting	M (10); U(40)	M (4); U(11)	M (4); U(31)	M (5); U(10)
	Pus formation	S (24); NS (26)	S (3); NS (12)	S (0); NS (35)	S (4); NS (11)
	**Laboratory Methods**				
	Histology performed	50	15	35	15
	Granuloma detected	39	4	0	1
	AFB detection	29	4	0	0
	Culture obtained	13	NA	ND	NA
	Biochemical	13	NA	ND	NA
	BCG vaccination	13	4	10	4
	Family history of TB	11	3	1	2
	**Demographics**				
	n	84	30		
	Age in years(mean ± SEM)	32 ± 2.6	28 ± 1.2		
	Gender male (%): female (%)	44(52):40(48)	18 (60): 12 (40)		
**PTB Cases**	Family history of TB	15	6		
	**Laboratory Methods**				
	Smear Microscopy	84	30		
	Radiology	84	30		
	Culture	68	ND		
	**Demographics**				
	n	130	20		
**Healthy controls**	Age in years(mean ± SEM)	27 ± 5	24 ± 3		
	Gender male (%): female (%)	62(77.5):18(22.5)	14 (70): 6 (30)		
	Family history Of TB	None	None		

Abbreviations: SEM: Standard error of the mean; C = Cervical; A = Axillary; SC = Supraclavicular; SM: Sub Mandibular; I = Inguinal; Su = Submental; P/RA = Pre/Retroauricular; SS = Supra sternal; L = Largest; S = Smallest; M = Matted; U = Unmatted; T = tender; N = Non-tender; ST = Suppurating; NS = Non- Suppurating.

### Independent Validation cohort

We recruited an independent validation cohort comprising PTB (n = 30), LNTB (n = 15) and HC (n = 20) inclusion and exclusion criteria remaining the same as elucidated in the enrollment of the discovery cohort from 2012 up to 2013. In addition, we recruited patients with enlarged lymph node which included Cancerous LAP (n = 35), LAP due to other causes which could not be classified clinically, also referred to as ‘Other Lymphadenopathies’ (n = 20) to compare the cytokine profile among LAP as well. The cancerous LAP samples were obtained from the Department of Radiotherapy, Vardhaman Mahavir Medical College and Safdarjung Hospital. The patient’s medical record was available and the patient enrolled in the study directly from the outpatient clinics and have not undergone any treatment for cancer. Informed written consent was obtained from all the patients.

### Measurement of serum cytokine levels

Three ml of venous blood was drawn and dispensed in BD vacutainers for serum (BD Franklin lakes, NJ, USA). Serum was obtained by centrifuging blood at 1600 x g for 30 minutes; sera were collected and stored at -80°C till further use. The Cytokines in collected serum were assessed using human cytokine ELISA kit purchased from Gen-probe (San Diego, CA) or Ray-biotech (Norcross, GA). Serum samples were serially diluted and assay was carried out as per manufacture’s instruction. The optical density readings in the linear range of the dose response curve were used for calculating the concentrations. The final concentrations (pg/ml) were obtained after multiplying the values by dilution factor. The sensitivity and range of cytokine detection ranged differently for different cytokines (pg/ml) as reported by the manufacturer and was taken into consideration for the analysis.

### Follow up of patients after ATT

Blood was collected from five subjects in 2013 who agreed to being followed up after they had undergone complete ATT and their lymph nodes had subsided significantly and not palpable in two of the cases. We assayed for the serum levels of IL-10 and IL-8 and TNF- β as described above.

### Individual Biomarker Evaluation

Descriptive statistics for the serum concentrations of each biomarker were obtained. The serum cytokine levels were expressed as mean ± standard deviation (SD). Overall, the data exhibited a departure from normal statistical distributions as indicated by the Shapiro-wilk test (S1 Table). Therefore for multiple comparisons of variables between groups with the non-parametric Kruskal-Wallis test followed by post hoc testing with Dunn’s was used. The filtered comparisons were then subjected to Mann –Whitney U test for pairwise comparison between groups. A two-tailed p value < 0.05 was considered statistically significant. Receiver operating characteristic (ROC) curves for predicting ability of the cytokines to classify LNTB versus PTB or HC cases were constructed for each individual cytokine. The criteria for relevance set to an area under the curve (AUC) value of > 0.65. All borderline p-values were considered so as to not discard any biomarker prematurely. Logistic regression on individual variables was performed to assess the effect of the clinical features on the outcome of cases as LNTB. All computations were done using SPSS version 16.0.

### Multivariate Panel selection and analysis

With the goal to include all candidate markers of potential value multivariate analysis was performed on an initial panel of biomarkers selected based on univariate analysis as described above. For this purpose, Random Forests supplemented with backward elimination procedure were employed to further eliminate the cytokines that may be unimportant in the presence of interactions using the R statistical software (R Core Team (2014). R: A language and environment for statistical computing. R Foundation for Statistical Computing, Vienna, Austria. URL http://www.R-project.org/). At each step of the backward elimination procedure, a random forest with 10,000 trees was run, and the least important variable was eliminated, and the procedure repeated until only one variable remained. The optimal set of variables was selected on the basis of minimal model errors obtained at each elimination.

### Validation cohort and modelling approaches

#### Conditional Inference tree modelling

A conditional inference tree model based on recursive partitioning approach was built using "Party" package in R. Each node represents a decision to go down one branch or the other depending upon the values depicted along the line connecting the successive nodes. Finally, each sample ends up in one of the terminal leaf classes. This was to access the classification accuracy of the cytokines in classifying people into correct classes. This approach is a supplement to linear models and also works well in the presence of nonlinear relationships in the data.

#### Multinomial modelling

Three cytokines from the multivariate panel obtained were subjected to an independent validation by enrolling a validation cohort as described above. This cohort included other lymphadenopathies. For LNTB cases we used the samples from both validation and discovery cohort (n = 63) while modelling. Since Random Forest models are ensembles of decision trees, they are difficult to interpret clinically. Therefore, multinomial modelling, which gives explicit formulae to calculate the probability of class membership was done in R using mlogit package.

#### Algorithm optimization

Like many clinical data, our data had imbalanced representation of samples in each class and multinomial models are known to perform poorly unless optimized. Two strategies for model training were employed and compared for optimization. The first employed balanced training sets using random sampling (20 each of Cancerous LAP and LNTB and 17 from Other LAP, as 20 was the number of cases in this group (the remaining samples formed the test set). The second approach employed proportionate sampling, i.e. selecting randomly sampled unbalanced training sets proportionate to the frequency in the original dataset.

### Data availability

The serum cytokine levels from all the subjects presented in this manuscript has been deposited in the Dryad Digital Repository and can be accessed at: http://dx.doi.org/10.5061/dryad.g91p3.

## Results

The clinical and pathological profile of the study groups is outlined in Table 1. All biomarkers were selected for their established role in the pathophysiology of TB infection. Only few of them have been tested as biomarkers for PTB and still fewer for LNTB. The subjects suffering from PTB or LNTB were checked clinically, radiologically and bacteriologically before inclusion in the study. Logistic regression revealed that age and sex had no effect on the outcome of disease (data not shown). In the discovery cohort the most common presentation in LNTB cases was enlarged cervical lymph nodes in 50% of the cases (25 cases) followed by Axillary lymph nodes (6 cases), Supraclavicular (5 cases), Submandibular (5 cases) and one each of inguinal, sub mental, pre/retro auricular lymph nodes. Few subjects had more than one site of involvement such as 2 cases had both cervical and axillary and 3 had both cervical and supraclavicular involvement. Only 60% of the FNA specimens from LNTB cases were positive for acid fast bacilli (AFB) and a mere 26% were culture positive. The positive cultures were confirmed biochemically to be *Mycobacterium tuberculosis* (MTB) (Table 1).

In the validation panel the most common presentation in the lymphadenopathy category was again the cervical lymph node (85%). These cases underwent the FNA but the culture information about the LNTB cases could not be obtained. The cancerous LAP cases enrolled in the study had been examined by FNAC and the reports were made available that were used to classify them into appropriate category. The other LAP cases comprised of cases that were subjected to FNAC and the histological report was not able to determine the cause of the enlarged lymph nodes (Table 1).

The individual biomarker evaluation is listed in Table 2. We enrolled the discovery cohort with the aim of identifying a set of biomarkers that could distinguish between LNTB and the most common presentation of TB, PTB and the HC. PTB was used as an additional comparison group to increase the specificity of any findings since comparing LNTB to only the HCs would also identify non-specific signatures related to general MTB infection. It was expected that such signatures that would be common to LNTB and PTB and could be eliminated. When compared to HC, serum levels of IL-8, IL-12, TNF-β,IL-6, IL-2 and IL-18 were significantly elevated in PTB cases and levels of IL-12, IL-10, IL-2, IL-6, IL-18 and TNF-βwere significantly elevated in LNTB cases (Table 2). The mean serum levels of IL-1β, IL-1RA did not show any significant variation as compared to HCs. Interestingly, the mean serum levels of IFN-γ and TNF-αwere lower in PTB when compared to HC. In LNTB, serum TNF-αlevels were similar but IFN-γ levels were lower (Table 2). This is not very surprising as India is an endemic country for TB with a high incidence and prevalence rate for TB, the population is generally deemed to be sensitized by the TB bacilli and cases may represent those with diminished primary response.

**Table 2:** Individual biomarkers tested in the discovery cohort and their efficacy in distinguishing the study groups.

Cytokine	Study groups	Mean serum cytokine level(pg/ml)*	Kruskal-Wallis p- value	Pairwise comparison	Mann-Whitney value	AUC (95% CI)	AUC p -values
	PTB	54 ± 68		PTB vs LNTB	0		
**IFN-γ**	LNTB	95 ± 99	0	PTB vs HC	0	0.487 (0.390–0.585)	0.807
	HC	156 ± 122		LNTB vs HC	0.003		
	PTB	39 ± 24		PTB vs LNTB	0.406		
**TNF-α**	LNTB	47 ± 73	0	PTB vs HC	0	0.376 (0.276–0.477)	0.017
	HC	47 ± 26		LNTB vs HC	0		
	PTB	81 ± 282		LNTB vs PTB	0		
**IL-8**	LNTB	212 ± 348	0	LNTB vs HC	0.262	0.792 (0.720–0.864)	0
	HC	42 ± 147		PTB vs HC	0		
	PTB	261 ± 438		LNTB vs PTB	0.312		
**IL-18**	LNTB	378 ± 473	0.009	LNTB vs HC	0.021	0.583 (0.470–0.696)	0.108
	HC	76 ± 177		PTB vs HC	0.006		
	PTB	96 ± 147		LNTB vs PTB	0.047		
**IL-12**	LNTB	73 ± 12	0	LNTB vs HC	0	0.761 (0.692–0.830)	0
	HC	48 ± 3.4		PTB vs HC	0		
	PTB	30 ± 19		PTB vs LNTB	0.001		
**IL-10**	LNTB	47 ± 37	0	PTB vs HC	0.73	0.743 (0.638–0.848)	0
	HC	23 ± 10		LNTB vs HC	0		
	PTB	4.7 ± 4.1		PTB vs LNTB	0.623		
**IL-4**	LNTB	3.4 ± 2.1	0.004	PTB vs HC	0.007	0.376 (0.281–0.471)	0.017
	HC	4.5 ± 2.1		LNTB vs HC	0.004		
	PTB	8.5± 12		LNTB vs PTB	0.004		
**IL-2**	LNTB	6.4 ± 0.76	0.001	LNTB vs HC	0.001	0.578 (0.479–0.677)	0.134
	HC	6.6 ± 1		PTB vs HC	0.584		
	PTB	103 ± 370		LNTB vs PTB	0.175		
**IL-1β**	LNTB	94 ± 184	0.297	LNTB vs HC	0.927	0.546 (0.440–0.652)	0.376
	HC	58 ± 24		PTB vs HC	0.141		
	PTB	196 ± 260		LNTB vs PTB	0.692		
**IL-1Ra**	LNTB	104 ± 81	0.86	LNTB vs HC	0.873	0.430 (0.342–0.519)	0.178
	HC	107 ± 87		PTB vs HC	0.555		
	PTB	1627 ± 1866		PTB vs LNTB	0.837		
**TNF- β**	LNTB	1866 ± 3030	0.064	PTB vs HC	0.176	0.608 (0.519–0.698)	0.036
	HC	1263 ± 2078		LNTB vs HC	0.006		
	PTB	76 ± 306		PTB vs LNTB	0		
**IL-6**	LNTB	62 ± 258	0	PTB vs HC	0	0.579 (0.488–0.670)	0.128
	HC	4.6 ± 21		LNTB vs HC	0		

* Values expressed as Mean± standard deviation;

Based on statistical evidence IL-8, IL-12, IL-10, IL-4, TNF-βIL-6, IL-18 and IL-2 were selected for further downstream analysis

A PTB vs LNTB comparison showed that the mean serum cytokine levels elevated in PTB as compared to LNTB were of IL-12, IL-6 and IL-2 and the mean serum levels elevated in LNTB as compared to PTB were of cytokines IL-8, IL-10 and IL-18. Looking at this comparison we can see a clear distinct panel of cytokines emerging which differ between the two conditions.

**Fig 2:**
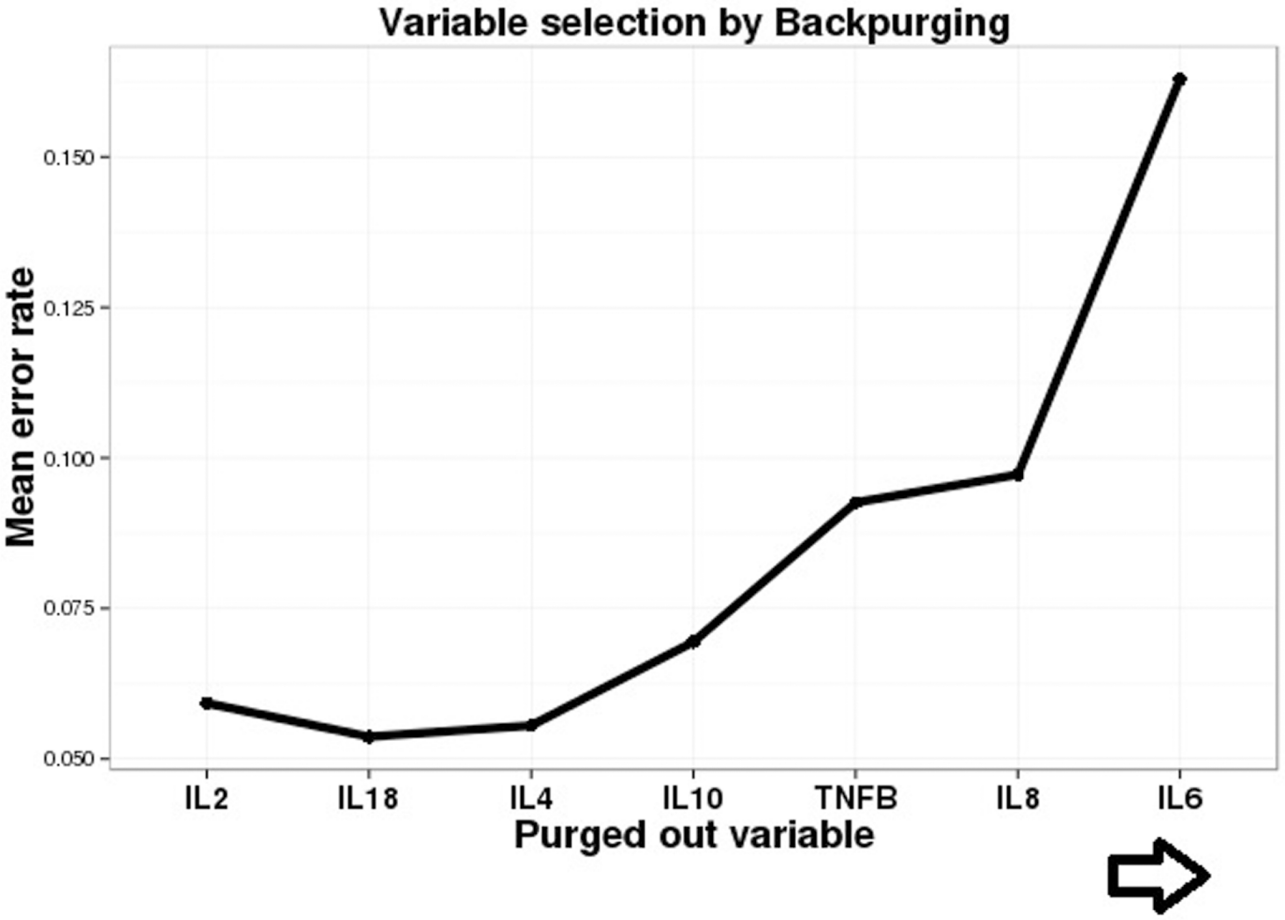
Backward elimination identifies the most important cytokines for the models. Random Forest backward elimination procedure to further eliminate the cytokines that may be unimportant in the presence of interactions. At each step of the backward elimination procedure, random forest with 10000 trees was run and variable importance and model errors were saved. The variable with the least importance was then purged out, and the procedure repeated until only one variable remained. The cytokines are not individually knocked out, but are sequentially knocked out from right to left as indicated by the arrow. Model errors were then plotted as a function of the order of the purged out variables. The minimum error was obtained with six cytokines (IL-4, IL-10, TNF-β, IL-8, IL-6 and IL-12) as shown in figure. The error rate increases after IL-18 is purged out, therefore implying that all the ones following IL-4 are important. One variable that remains in the end and is not shown in the figure is IL-12.

**Fig 3:**
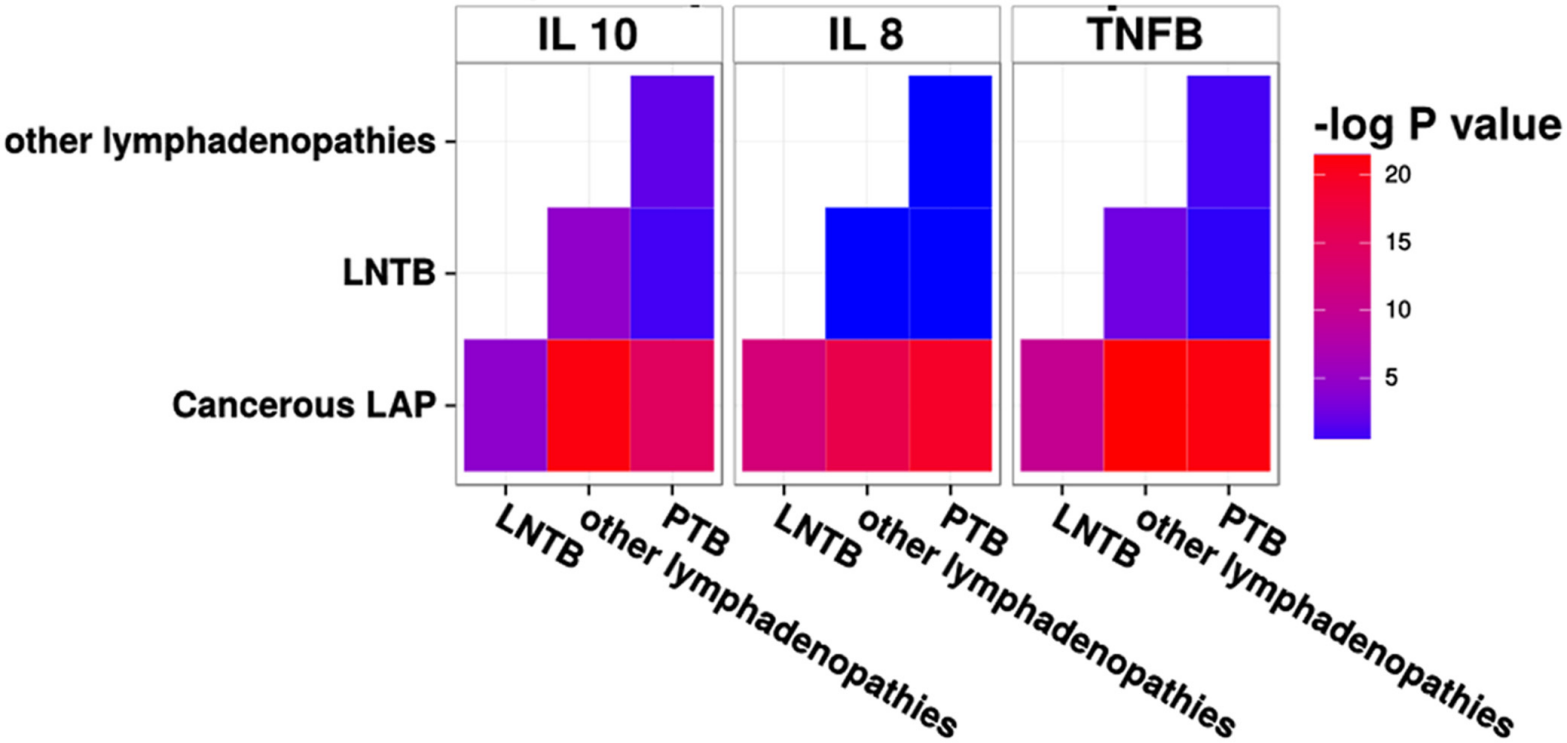
Heatmap of pairwise discriminatory power of individual cytokine in the validation cohort. Each panel is representing a cytokine. Phenotypes are on x and y axes. Each combination of phenotypes is filled by -log p value obtained from Tukey’s posthoc testing after ANOVA. If any combination of two phenotypes is filled with red/pink, then the particular cytokine is significantly different between the two groups and thus may help in distinguishing them.

**Fig 4:**
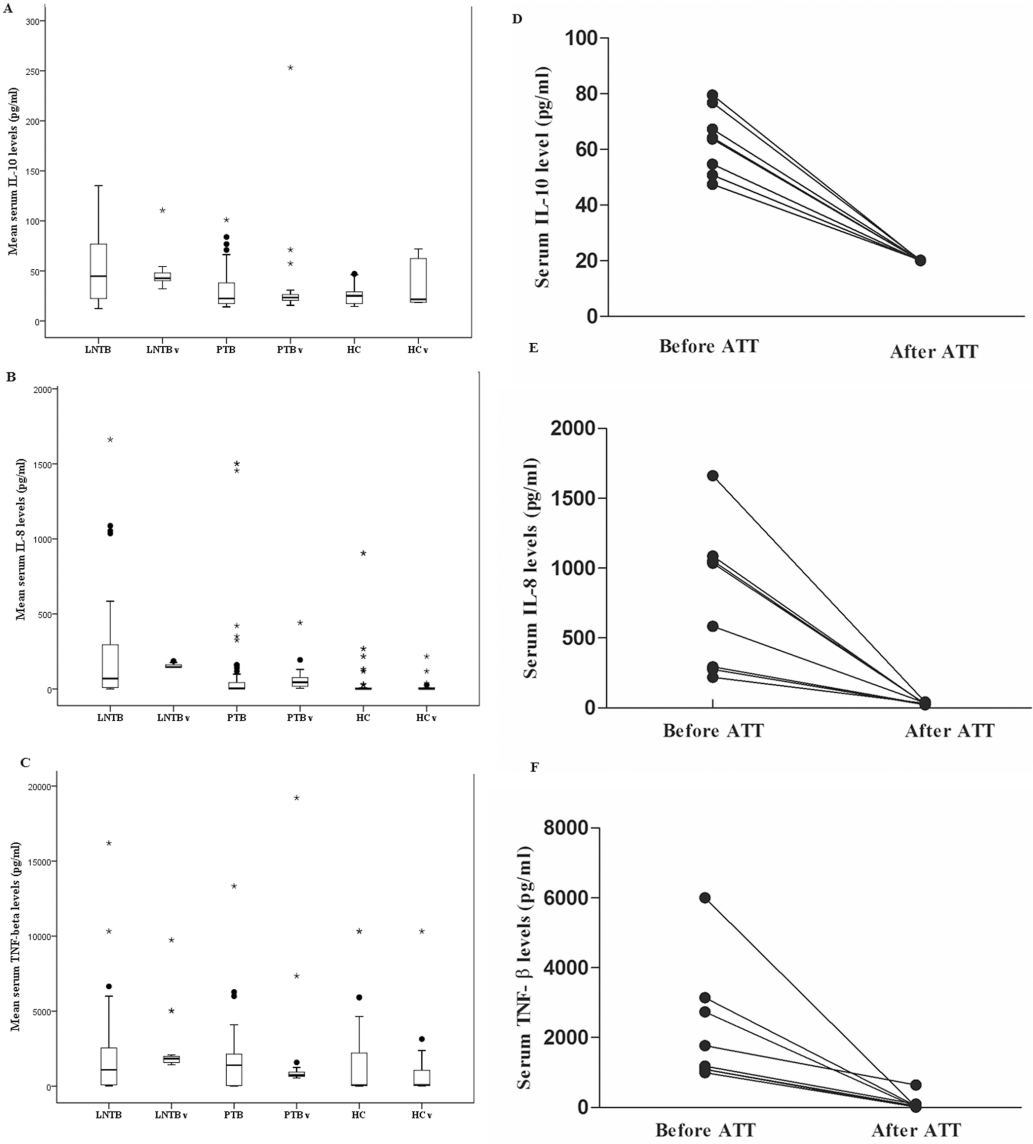
Comparison of mean serum levels of IL-10, IL-8 and TNF-β between the discovery and validation cohorts and the change in levels after treatment with ATT. “v” added to the group name indicates the value from validation panel. Kruskal-Wallis test followed by Mann-Whitney U test was used to compare the groups as the data was not normally distributed. The results indicated that no significant difference in the mean serum cytokine levels was seen among the derivation and validation panel among the groups (A) for IL-10 [(LNTB vs LNTBv (p = 0.928), PTB vs PTBv (p = 0.265), HC vs HCv (p = 0.215)] and (B) IL-8 [(LNTB vs LNTBv (p = 0.197), HC vs HCv (p = 0.862) except for PTB vs PTBv (p = 0.000),] but for (C) TNF-β LNTB vs LNTBv (p = 0.016), PTB vs PTBv (p = 0.031) except HC vs HCv (p = 0.914)] showed significant difference between the groups, (D-F) Showing the change in levels of the investigated cytokines after completion of ATT. Each dot represent an individual and the change in their corresponding levels (n = 8). A statistically significant reduction in the serum levels was observed. (D) The mean serum levels for the eight followed up individuals went from being 63.06 + 11.6 pg/ml to 20.11 ± 0.08 pg/ml for IL-10 (p = 0.0002), (E) from 776.4 ± 515 pg/ml to 30.17 ± 6.8 pg/ml for IL-8 (p = 0.0002) and (F) from 2251 ± 1721 to 110.5 ± 217. 5 pg/ml for TNF-β p = 0.0009).

The ROC analysis done for classifying LNTB correctly as compared to PTB and HC gave significant area under the curve (AUC) for IL-8, IL-12, IL-10 and TNF-β with borderline AUC for IL-18, IL-2 and IL-6 (Table 2, S1 Fig). Combined exploratory analysis and the ROC curves helped us select 8 out of 12 cytokines which include: IL-8, IL-12, IL-10, IL-4, TNF-β, IL-6, IL-18 and IL-2. Since these cytokines may still not be independent of each other, the obtained panel was further reduced by feature selection procedures using Random Forests. With Random forest back purging the minimum error was obtained with 6 cytokines (IL-4, IL-10, TNF-β, IL-8, IL-6 and IL-12) as shown in Fig 2. Out of these six cytokines which classified LNTB with least error among the three investigated classes of the discovery cohort, namely PTB, LNTB and HC, we eliminated IL-6 since it has been widely studied as a biomarker for PTB [11] and was elevated in our study as well for PTB cases. While LNTB had an intermediate level of IL-6 that permits classification, clinical ambiguity is likely. IL-4 and IL-12 have also been associated with the outcome of PTB [11] and have similar problems. So only three cytokines IL-10, IL-8 and TNF- β were selected further and verified in an independent validation cohort, keeping in mind resource limited settings and need for a simple signature. The reason for enrolling a new validation cohort with mixed lymph node pathologies was twofold. First, to confirm the observed serum cytokine levels among PTB, LNTB and HC. Second, the real clinical question would be the nature of lymphadenopathy rather than whether a patient has lymphadenopathy (which is evident most of the times). A pairwise comparison between the levels of the three cytokines among the groups of the validation panel showed that IL-10 could distinguish between most of the studied groups i.e. cancerous LAP, Other LAP, PTB, while IL-8 and TNF-βcould distinguish between all other groups except for between LNTB vs Others LAP (Fig 3). When the serum levels of these cytokines were compared between corresponding groups of discovery and validation cohort no statistically significant difference was observed for IL-10 and IL-8 (Fig 4A and 4B). TNF-β showed difference in LNTB and PTB groups except for HC (Fig 4C).

**Fig 5:**
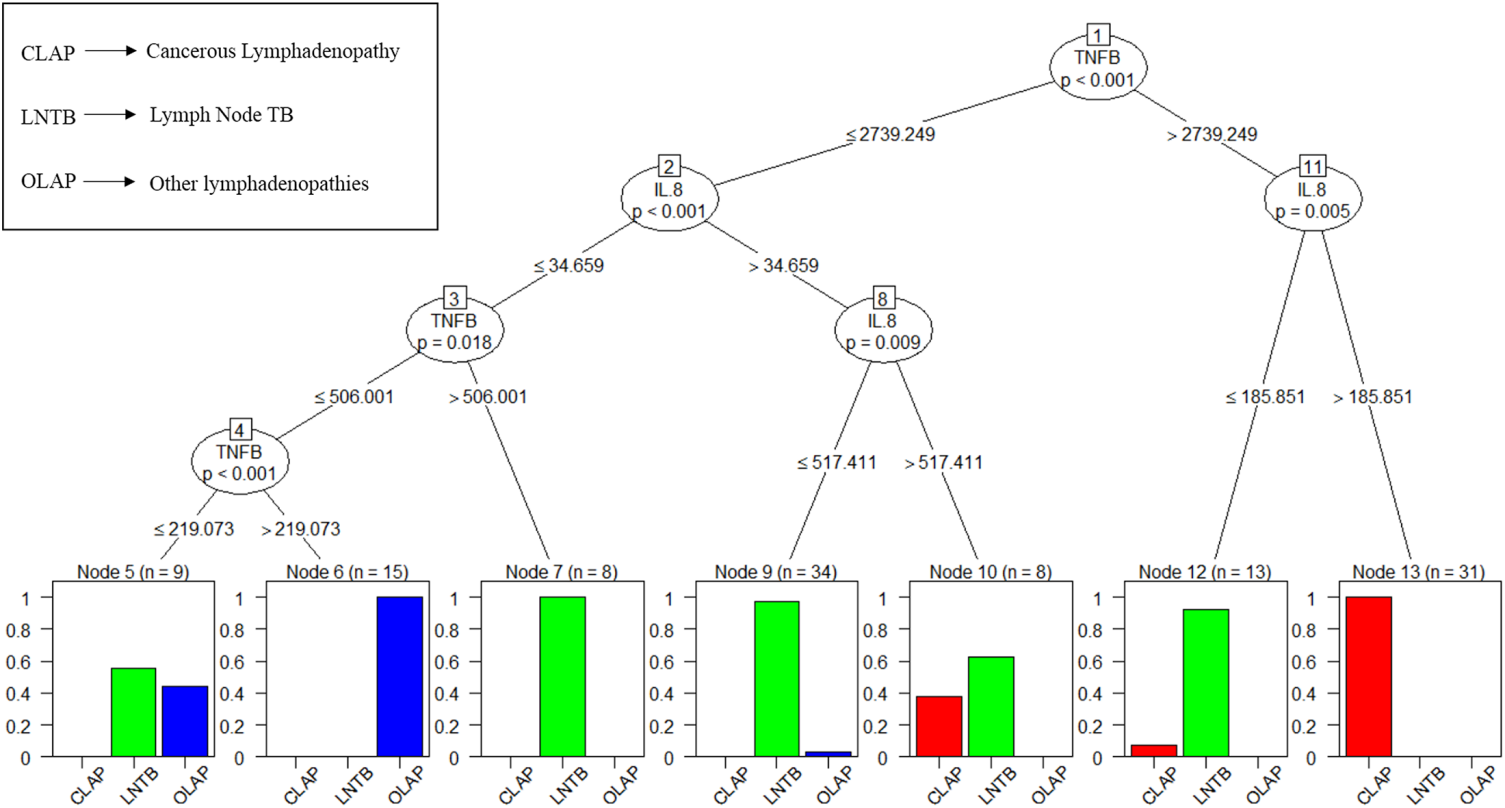
Conditional inference tree as applied to cytokine concentrations from all lymphadenopathies. For each inner node, the Bonferroni adjusted P-values are given. Concentrations are in ng/ml. For example in the terminal node 5, nine samples fulfilled the sequential criteria: (TNF- β < 2739.25) and (IL-8 < 34.659) and (TNF- β < 506) and (TNF β < 219.07). The bars represent probabilities of belonging to respective classes, in this case 60% probability of patient being cancerous, 40% probability of the patient being Other LAP, and 0% chance of being LNTB. Decision tree was made on full data and since the multinomial logistic model was validated on a held-out set.

To determine whether the combination of the three cytokines correctly classifies the lymphadenopathies, we used decision trees to visualize the classification (Fig 5). The decision tree model showed a 100% accuracy for classifying “LNTB’ and 88% for ‘cancerous LAP’ and 75% for ‘Other LAP’ (S2 and S3 Tables). While it was possible to use simple single cytokine cutoffs to separate the LNTB, HC, and PTB groups in both cohorts, non-tuberculous lymphadenopathy posed a more complex challenge, multinomial modelling was therefore done using these three biomarkers (IL- 8, IL- 10 and TNF-β) to be able to differentiate between the different classes of studied LAP as compared to LNTB. The sample size used was LNTB (n = 63); Cancerous LAP (n = 35) and Other LAP (n = 20) (S4 Table). The two strategies namely using a balanced and using proportionate sampling used are illustrated (in the methods, Fig 1). While testing the model to get all the distinguishing information the models were tested by using LNTB and cancerous LAP as reference groups (Table 3).

**Table 3:** Summary of model characteristics for classifying LNTB from Cancerous LAP and Other LAP.

	Models	Multinomial Logistic regression with Balanced sampling				Multinomial Logistic regression with proportionate sampling			
Outcome variable /Reference category	Alternative category: Predictor variable	Estimate^@^	t-value^#^	SE^$^	P value (t)^$$^	Estimate	t-value	SE	P value (t)
	Cancerous LAP: (intercept)	-4.9283908	1.7983352	-2.7405	0.00613**	-5.6034689	1.4979909	-3.7407	0.000184***
	other LAP: (intercept)	4.6111119	1.7001833	2.7121	0.00669**	1.9151255	0.7471673	2.5632	0.010372*
	Cancerous LAP: IL-10	0.0386316	0.0194854	1.9826	0.04741*	0.0280509	0.0137314	2.0428	0.04107*
**LNTB**	other LAP: IL-10	-0.1300263	0.0712198	-1.8257	0.0679^	-0.0459957	0.0299497	-1.5358	0.124597
	Cancerous LAP: IL-8	0.0026057	0.0011965	2.1778	0.02942*	0.0034938	0.0010735	3.2545	0.001136**
	other LAP: IL-8	-0.0032404	0.0108226	-0.2994	0.76463	-0.006708	0.0067589	-0.9925	0.320966
	Cancerous LAP: TNF-β	0.0002186	0.0001356	1.6118	0.107	0.0003438	0.0001161	2.9621	0.003055**
	other LAP: TNF-β	-0.0020728	0.0013072	-1.5856	0.11282	-0.0016423	0.0007593	-2.1628	0.030559*
	LNTB: (intercept)	4.9283908	1.7983352	2.7405	0.00613**	5.6034689	1.4979909	3.7407	0.000184***
	Other LAP: (intercept)	9.5395027	2.4703872	3.8615	0.00011***	7.5185945	1.6701271	4.5018	0.00000674***
	LNTB: IL-10	-0.0386316	0.0194854	-1.9826	0.04741*	-0.0280509	0.0137314	-2.0428	0.04107*
**Cancerous LAP**	Other LAP: IL-10	-0.1686579	0.0739352	-2.2812	0.02254*	-0.0740465	0.0329216	-2.2492	0.024501*
	LNTB: IL-8	-0.0026057	0.0011965	-2.1778	0.02942*	-0.0034938	0.0010735	-3.2545	0.001136**
	Other LAP: IL-8	-0.0058462	0.0108884	-0.5369	0.59133	-0.0102018	0.0068429	-1.4909	0.135997
	LNTB: TNFB -β	-0.0002186	0.0001356	-1.6118	0.107	-0.0003438	0.0001161	-2.9621	0.003055*
	Other LAP: TNF-β	-0.0022914	0.0013144	-1.7434	0.08127^	-0.001986	0.0007683	-2.5851	0.009736*

^@^ logistic coefficient (estimate) for each predictor variable for the alternate category (Cancerous LAP and other LAP) while trying to predict the reference category (LNTB) or predicting the reference category (Cancerous LAP) with alternate category for this comparison bring (LNTB and other LAP). The “estimate” is the expected amount of change in the “logit” (the odds of being in the category of the outcome variable which has been specified) for each unit change in the predictor (the respective cytokine levels). The closer the estimate is to 0 the less influence the predictor has in predicting the logit. Note in most of the comparison the estimate value is very close to zero meaning that alternate category is easily distinguished from the reference category based on the predictor cytokine for that comparison.

^#^ t- value is the value for t- test for significance

^$^ SE: Standard error

^$$^ P value (t) is it’s respective p–value.

Significance codes:

‘***’ 0.001;

‘**’ 0.01,

‘*’ 0.05,

‘^’ 0.1,

‘ ’ 1.

Both the models were showed a better fit from the baseline with the proportionate model showing log-likelihood ratio = -38.645, McFadden R^2: 0.58802, χ^2^ = 110.32, p < 2.22e-16 and the balanced sampling model showing log-likelihood ratio = -22.019, McFadden R^2: 0.64747, χ^2^ = 80.882, p = 2.348e-15. The model from balanced sampling with LNTB as reference group showed that serum levels of IL-10 (p = 0.04741) and IL-8 (p = 0.02942) were significant predictors for distinguishing between LNTB and Cancerous LAP while only IL-10 (p = 0.0679) could distinguish it from other LAP (Table 3). IL-10 (p = 0.02554) and TNF-β (p = 0.08127) could distinguish between cancerous LAP vs other LAP (Table 3). For the proportionate sampling with LNTB as the reference group all three IL-8 (p = 0.001136), TNF-β (p = 0.003055) and IL-10 (p = 0.04107) were found to be predictive for LNTB versus Cancerous LAP. TNF-β was significant for predicting other LAP from LNTB (p = 0.030559). For Cancerous LAP as reference group TNF-β was significant for predicting other LAP (p = 0.009736). IL-10 was also significant predictor of others from cancerous LAP (p = 0.024501) (Table 3). The probability equations for both the sampling methods used for prediction of classes using the three cytokines are depicted in Table 4.

**Table 4:** Showing the equation for the prediction of probability (π) of the three lymphadenopathy classes using mean serum levels of IL-10, IL-8 and TNF-β.

Sampling method	Classes	Probability (π) of
	LNTB	= 1/1 + exp (− 5.6034 + 0.028 *IL* 10 + 0.0034 *IL* 8 + 0.0003*TNFβ*) + exp (1.915 − 0.046 *IL* 10 − 0.0067 *IL*8 − 0.0016 *TNFβ*)
**Proportionate sampling**	Cancerous LAP	= + exp (− 5.6034 + 0.028 *IL* 10 + 0.0034 *IL* 8 + 0.0003 *TNFβ*)/1 + exp + exp (− 5.6034 + 0.028 *IL* 10 + 0.0034 *IL*8) + exp (1.915 − 0.046 *IL*10 − 0.0067 *IL* 8 − 0.0016 *TNFβ*)
	Other LAP	= exp (1.915 − 0.046 *IL* 10 − 0.0067 *IL* 8 − 0.0016 *TNFβ*)/1 + exp (− 5.6034 + 0.028 *IL*10 + 0.0034 *IL*8 + 0.0003 *TNFβ*) + exp (1.915 − 0.046 *IL* 10 − 0.0067 *IL* 8 − 0.0016 *TNFβ*)
	LNTB	= 1/(1 + exp {− 4.9254 + 0.0386 *IL* 10 + 0.0026 *IL* 8 + 0.0002 *TNFβ*}) + exp (4.6111 − 0.13 *IL* 10 − 0.0032 *Il* 8 − 0.0021 *TNFβ*)
**Balanced sampling**	Cancerous LAP	= exp (−4.9254+0.0386*IL*10+0.0026*IL*8+0.0002*TNFβ*)/(1+exp {4.6111−0.13*IL*10−0.0032*IL*8−0.0021*TNFβ*})+exp (4.6111−0.13*IL*10−0.0032*IL*8−0.0021*TNFβ*)
	Other LAP	= exp (4.6111 − 0.13 *IL* 10 − 0.0032 *IL* 8 − 0.0021 *TNFβ*)/1 + exp (− 4.9254 + 0.0386 *IL* 10 + 0.0026 *IL* 8 + 0.0002 *TNFβ*) + exp (4.6111 − 0.13 *IL* 10 − 0.0032 *IL* 8 − 0.0021 *TNFβ*)

While balanced sampling achieved an 81.96% overall accuracy for classification between lymphadenopathies, the proportionate sampling method achieved an overwhelming 95.83% in the test set. The misclassification error in the test set was higher for the balanced sampling for LNTB (20.3%) as compared to an acceptable (7.6%) in the proportionate method. The cancerous LAP and others were well classified using the 3 cytokines in the model (Table 5). The confusion matrix and the data about the training and test set is supplied as appendix (S5–S8 Tables). From this it can be concluded that the proportionate sampling equations can be applied in clinical settings to predict the probability of the LAPs based on the three cytokines. For clinicians’ ease of use, we have included a MS-Excel sheet with a macro to calculate the probabilities of the three classes, using the equations presented in Table 4. This prediction can be obtained by pasting the cytokine levels into respective cells followed by executing the macro using shortcut keys ctrl+w. Instructions are also included in the excel sheet (S1 Sheet). Although we have presented both the decision tree model (Fig 5, S9 Table) and the multinomial model, with a good concordance (S10 Table); the multinomial model was slightly more accurate, especially for the classification of ‘Other LAP’. We suggest using the MS-Excel sheet with the multinomial model to predict the classes, which would be easier and possibly more accurate for clinicians.

**Table 5:** Overall performance characteristics of the multinomial models build using IL-10, IL-8 and TNF-β levels as classifying variables.

Model	Overall accuracy	Studied groups	Misclassification Error *	Sensitivity*	Specificity*	PPV	NPV
		LNTB	20.3	79	89	94	64
Multinomial Logistic regression with a Balanced sampling	81.9%	Cancerous LAP	13.3	87	89	72	95
		Other LAP	0	100	93	42	100
		LNTB	7.6	92	100	100	91
Multinomial Logistic regression with proportionate sampling	95.83%	Cancerous LAP	0	100	94	87	100
		Other LAP	0	100	100	100	100

* The data expressed as percentage. PPV: Positive predictive value; NPV: Negative Predictive value.

Attempt to follow-up on these LNTB cases to determine whether the levels of the tested cytokines recede after successful treatment resulted in only five of the subjects who agreed to be a part of it. The level of all the three cytokines had receded significantly and was now comparable to that of healthy controls for IL-10 (Fig 4D) and IL-8 (Fig 4E). Surprisingly, for TNF-β the recorded serum level was far lower than the HCs (Fig 4F).

## Discussion

A diagnostic method should be rapid, less invasive, minimally painful to the patient and discriminatory enough. Detecting biomarkers of immunological value in the serum of patients concedes to the aforementioned notion. Until date the diagnosis of LNTB relied heavily on the conventional FNA, smear microscopy and culture of the aspirates. But these techniques have their limitations as the samples are often paucibacillary and an accurate diagnosis often eludes the clinician. Apart from the conventional techniques, few other diagnostic methods that have been tried for LNTB includes Nucleic acid amplification tests (NAATs) for mycobacteria [19–20] and Interferon-gamma release assay (IGRAs) [21] with varying degrees of success. IGRAs have been tried primarily in PTB [21] and recently in EP-TB [22–23]. It has been shown that performance of IGRAs varies in different population [24–26]. A higher TNF-α and IFN-γ were found in HCs in this study to that effect Rangaka et al[21] concluded as IFN-γ could indicate MTB sensitization (rather than disease) it might not be adequate to predict active tuberculosis, especially a high TB burden and rates of re-infection. As for the sensitivity and specificity of the abovementioned assays for diagnosis of EP-TB; NAATs have shown a sensitivity ranging from 43–84% and specificity from 75–100% [4]. IGRAs in a study by Song and colleagues [22] showed a modest performance of 69% sensitivity and 82% specificity in a sample size of 48 cases of which only 33 were EP-TB positive. Kim et al [23] report IGRAs to be 92% sensitive and 80.2% specific with a sample size of 25 EP-TB cases. There is thus an apparent void which could be taken over by new promising immunological biomarkers. Studies on serum cytokine response in EP-TB by Juffermans et al [12], Verbon et al [13] and Hasan et al [14] considered different EP-TB manifestations together for analysis (see S1 Appendix for breakdown) and observed no difference in the cytokine levels between EP-TB and PTB patients as compared to HC. No particular extra-pulmonary manifestation was studied for in depth resulting in lack of any signature biomarker for any of the EP-TB.

The serum cytokine response in LNTB that had never been investigated systematically as a cohort study. Cytokines being key mediators of immune response shape up the host’s ability to fight the pathogen successfully and do not function independent of each other but in fact are involved in a complex in-vivo dynamics governing the outcome of infection. Therefore, a dysfunctional cytokine network should be investigated in TB rather than up or down-regulation of only a certain cytokine [14]. The cytokine profile for PTB patients has been reviewed for comparison (the comparison with references supplied as S2 Appendix), so we focus our discussion on LNTB as that was our primary aim. Based on the cytokine profile we propose that the role of cytokines typified here by high serum IL-10, IL-8 and TNF-β becomes evident in affliction of localized area and response such as lymph nodes. The basic immune response in the lymph nodes is containment of infection as the patients with LNTB did not present with classical clinical symptoms of tuberculosis. Lymph nodes represent an ideal environment for generation of immune response due to the presence of all immune cells in close vicinity (see S3 Appendix). To the best of our knowledge, this is the first study from the region to explore such vast panel of the cytokines in PTB and LNTB to give a picture of immune pathogenesis and identify host serum biomarkers. Based on our results we could identify a definitive role for serum IL-10, IL-8 and TNF-β for LNTB which showed better sensitivity and specificity as compared to the rest of the cytokines in the panel. Our results have several important clinical implications. First, as a putative new test based on detection of these cytokines in LNTB cases, it offers a high level of certainty based on presented data. In our study, we had an equal proportion of AFB + and–individuals with granulomatous findings in 80% of the cases but the cytokine level was found to be elevated irrespective of the clinical findings. So, this new set of immunological markers can clearly distinguish LNTB cases and can score where AFB positivity and granulomatous findings elude the clinician. The MS-Excel sheet based on the probability equations, can be used by physician in their clinical decision making and step wise management of LNTB patients. As evident from the follow-up samples these biomarkers can also be applied to judge the clinical endpoint in the patient. The seemingly unusual lowering of TNF-β could be attributed to the chemotherapy that the patient underwent. TNF-β axis is closely related to TNF- α axis [18] and as in the HCs the levels of TNF-α were significantly higher the levels of TNF-β were also high. Following anti-tubercular therapy there must have been resolution of the heightened inflamed state and thus significant lowering of TNF-α and resultant lowering of TNF-β. Although an attempt was made to plan and execute a comprehensive study, it was not without limitations, which are discussed subsequently. Only a small number of people who completed their ATT could be followed up. As can be observed from the data that there were modest differences in the serum levels of certain cytokines between the study groups, such as IL-4. These modest differences were uncovered and realized only through a combination of statistics and machine learning approaches, which would have been lost otherwise. This supports the application of such statistical manipulations for uncovering minute differences in biomarker discovery [27]. The study population, both the discovery and validation cohorts, were from north India which makes the study limited in population demographics. Studies have shown that diagnostic test performance may vary between populations, as is the case with NAATs [4] and IGRAs [22–23]. It could be argued that the relatively younger population (means of age range from 27 to 32 years) among various study groups limits generalization to older subjects as it has been shown that some of the inflammatory cytokines increase or decrease in the serum of healthy subjects as they age [28]. Interestingly the panel that we have studied here, i.e. IFN-γ, IL-6, IL-8, did not show any major changes with age [28] so these results could be applicable to the elderly. Overall, based on the results obtained here, we propose that these signature biomarkers could be further explored in larger prospective studies from various regions of India and other countries to validate our observation.

## Supporting Information

S1 Appendix Sample size distribution of various studies that included various EP-TB under the same banner. (DOCX)

S2 Appendix Cytokine profile in pulmonary tuberculosis patients. (DOCX)

S3 Appendix Cytokine profile of LNTB as observed in the study. (DOC)

S1 Fig Showing the ROC curves for LNTB vs PTB and Healthy controls. (TIF)

S1 Sheet Excel sheet embedded with probability calculations to be used by clinicians. (XLSM)

S1 STROBE Checklist STROBE Checklist. (DOC)

S1 Table Showing the results of the test for normality of the data. (DOCX)

S2 Table Confusion matrix of testing set for classification of the LAP classes using the decision tree model. (DOCX)

S3 Table Class wise accuracy for the decision tree model. (DOCX)

S4 Table Total sample size in each of the three classes used for multinomial modelling. (DOCX)

S5 Table Sample size for modelling using balanced sampling. (DOCX)

S6 Table Sample size for modelling using proportionate sampling. (DOCX)

S7 Table Confusion matrix of testing set for classification of the LAP classes using the model built with balanced training set. (DOCX)

S8 Table Confusion matrix of testing set for classification of the LAP classes using the model built with proportionate training set. (DOCX)

S9 Table Decision tree model original and predicated class. (DOCX)

S10 Table Concordance of the prediction between the decision tree and the multiple logistic regression models. (DOCX)
