# Supplementary material for: Establishment of Elevated Serum Levels of IL-10, IL-8 and TNF-β as Potential Peripheral Blood Biomarkers in Tubercular Lymphadenitis: A Prospective Observational Cohort Study
Source: PLoS One. 2016 Jan 19;11(1):e0145576. doi: 10.1371/journal.pone.0145576 (PMC4718686; doi:10.1371/journal.pone.0145576)
Supplement: S1 Appendix — (DOCX) [file pone.0145576.s001.docx]

**S1 Appendix**

**Sample size distribution of various studies that included various EP-TB under the same banner:**

A study on serum cytokine response in EP-TB by Juffermans and colleagues [1] considered different EP-TB manifestations together for analysis ( n=32: LNTB = 8, Pleural effusion = 12, bone and joints = 6, meninges = 2, gastro-intestinal specimen = 2, soft tissue=2, disseminated disease =2) and observed no difference in the cytokine levels between EP-TB and PTB patients as compared to HC. Another study by Verbon and colleagues [2] followed the same approach and their EP-TB cases (n = 36) included (Pleural effusion =11; Lymph node TB = 9; Soft tissue infection = 2; TB Meningitis=3; Gastro intestinal TB=2; bone and joints TB =6; disseminated disease=3). A study by Hasan and colleagues [3] analyzed 38 EP-TB cases divided into severe (n = 20) and less severe (n = 18) EP-TB cases but they also considered the different manifestations together. The less severe cases included 17 lymphadenitis and 1 genito-urinary TB, and the rest 20 cases of severe EP-TB disease included TB meningitis, abdominal TB, spinal TB, bilateral pleural effusion and military TB.

**References:**

1 Juffermans NP , Verbon A , van Deventer SJ , van Deutekom H , Speelman P , van der Poll T .Tumor necrosis factor and interleukin-1 inhibitors as markers of disease activity of tuberculosis. *Am J Respir Crit Care Med* 1998; 157: 1328–31.

2 Verbon A , Juffermans NP , Van Deventer SJ , Speelman P ,Van Deutekom H, Van Der Poll T .Serum concentrations of cytokines in patients with active tuberculosis TB and after treatment. *Clin Exp Immunol* 1999; 115:110–3.

3 Hasan Z, Jamil B, Khan J, Ali R, Khan MA, Nasir N et al. Relationship between circulating levels of IFN-gamma, IL-10, CXCL9 and CCL2 in pulmonary and extrapulmonary tuberculosis is dependent on disease severity. *Scand J Immunol* 2009; 69 : 259–67.
