# Supplementary material for: Establishment of Elevated Serum Levels of IL-10, IL-8 and TNF-β as Potential Peripheral Blood Biomarkers in Tubercular Lymphadenitis: A Prospective Observational Cohort Study
Source: PLoS One. 2016 Jan 19;11(1):e0145576. doi: 10.1371/journal.pone.0145576 (PMC4718686; doi:10.1371/journal.pone.0145576)
Supplement: S2 Appendix — (DOCX) [file pone.0145576.s002.docx]

**S2** **Appendix**

**Cytokine profile in pulmonary tuberculosis patients**

The cytokine profile for pulmonary tuberculosis patients that has emerged is as follows: IFN–γ was found to be elevated in TB patients in a number of studies [4, 6,10,12,16 –17, 19 –20, 25 – 26, 29]. In our study, as compared to PTB cases IFN –γ was found to be higher in HC. TNF–α has shown a mixed profile of being high [5,8,14–15,23–24] or low [17,20,27] or unchanged in some studies [16,19] and even was undetectable in one study 5. In our study serum TNF–α was low in patients’ sera as compared to HCs as had also been reported by Atkas and colleagues [27]. Serum IL–12 had been studied in a limited number of reports [1, 10, 17, 19, 29] and was found to be higher in two studies [17, 19] and low in one study [10]. In our study the mean serum IL–12 levels were significantly high in PTB. Reports on serum IL–8 levels are equivocal. While some reports show no change [19, 22] and a few reports show high serum levels in PTB [9] , in our study, the mean serum IL–8, though higher did not differ significantly between cases and controls. Serum IL–18, a synergistic potent inducer of IFN–γ in conjunction with IL–12, has been reported to be elevated in tuberculosis in some reports [6, 29, 30] but in our study although the levels were higher, the difference was not significant. Serum levels of other important cytokines such as IL–1β has been reported to be higher in PTB [7, 12, 14] but others [5] have not found even detectable levels of the cytokine. Although we found high levels of serum IL–1β as compared to HC, the difference was not significant. Serum IL–1Ra has been reported to be consistently up regulated in PTB as compared to HC in some studies [5, 7, 14, 22]. However, it was not found to be significantly elevated in the present study. Consistent with the equivocal reports on limited serum detection of IL–4 in PTB majority of researchers have reported low [10,27] or no difference in the serum levels [17,19–20,23] with only one study [31] reporting high serum level of IL–4.We also did not find any significant change in the level of serum IL–4 in the present study. The mean serum IL–2 also spanned from high [2, 11], no change [12, 19] to lower [27] in other studies, but was not found to be significantly different between cases and controls in the present study. Some studies report high serum IL–10 levels [10, 25–26, 29] however, others have reported no difference between cases and controls [20], and in our study no difference in serum IL–10 level between cases and controls was observed. TNF–β levels have not been reported in PTB and we report for the first time significantly elevated level of this cytokine in PTB as compared to healthy controls.

The picture of immunopathogenesis that emerges is that recognition of Mycobacteria by the phagocytic cells leads to the production of cytokines which further starts a cascade of regulation and cross–regulation. Auto induction and secretion of major pro–inflammatory cytokines such as TNF–α, IFN–γ and IL–1β by macrophages and/or dendritic cells try to fight off the bacteria, which is aided by IL–18 and TNF–β. The main inducer of IFN–γ, IL–12 has been hailed to be efficacious at low levels [32] .In our cases from north India the mean serum IL–12 was found to be very high. It is possible that here IL–12 might play a dual role: of initially inducing IFN–γ but over induction due to high levels of IL–12 may itself be hampering its production.

**References:**

1 Juffermans NP , Verbon A , van Deventer SJ , van Deutekom H , Speelman P , van der Poll T .Tumor necrosis factor and interleukin-1 inhibitors as markers of disease activity of tuberculosis. *Am J Respir Crit Care Med* 1998; 157: 1328–31.

2 Verbon A , Juffermans NP , Van Deventer SJ , Speelman P ,Van Deutekom H, Van Der Poll T .Serum concentrations of cytokines in patients with active tuberculosis TB and after treatment. *Clin Exp Immunol* 1999; 115:110–3.

3 Hasan Z, Jamil B, Khan J, Ali R, Khan MA, Nasir N et al. Relationship between circulating levels of IFN-gamma, IL-10, CXCL9 and CCL2 in pulmonary and extrapulmonary tuberculosis is dependent on disease severity. *Scand J Immunol* 2009; 69 : 259–67.

4 R. Ribeiro–Rodrigues, T. Resende Co, J.L. Johnson, F. Ribeiro, M. Palaci, R.T. Sá, E.L. Maciel, F.E. Pereira Lima, V. Dettoni, Z. Toossi, W.H. Boom, R. Dietze, J.J. Ellner, C.S. Hirsch, Sputum cytokine levels in patients with pulmonary tuberculosis as early markers of mycobacterial clearance, Clin Diagn Lab Immunol. 9 2002 818–23.

5 N.P. Juffermans , A. Verbon , S.J. van Deventer , H. van Deutekom , P. Speelman , T .van der Poll , Tumor necrosis factor and interleukin–1 inhibitors as markers of disease activity of tuberculosis, Am J Respir Crit Care Med. 157 1998 1328–31.

6 G. Yamada, N. Shijubo, K. Shigehara, H. Okamura, M. Kurimoto, S. Abe, Increased levels of circulating interleukin–18 in patients with advanced tuberculosis, Am J Respir Crit Care Med. 161 2000 1786.

7 T.C. Tsao , J. Hong , L.F. Li , M.J. Hsieh , S.K. Liao , K.S. Chang ,Imbalances between tumor necrosis factor–alpha and its soluble receptor forms, and interleukin–1beta and interleukin–1 receptor antagonist in BAL fluid of cavitary pulmonary tuberculosis, Chest. 117 2000 103–9.

8 A. Wanchu , A. Bhatnagar , J. Talreja , S. Sapra , B.S. Suryanarayana , P. Suresh , Immunophenotypic and intracellular cytokine profile of Indian patients with tuberculosis with and without human immunodeficiency virus co–infection, Indian J Chest Dis Allied Sci. 51 2009 207–11.

9 N.P. Juffermans , A. Verbon , S.J. van Deventer , H. van Deutekom , J.T. Belisle , M.E. Ellis , P. Speelman , T. van der Poll , Elevated chemokine concentrations in sera of human immunodeficiency virus HIV–seropositive and HIV–seronegative patients with tuberculosis: a possible role for mycobacterial lipoarabinomannan, Infect Immun. 67 1999 4295–7.

10 A. Verbon , N. Juffermans , S.J. Van Deventer , P. Speelman , H. Van Deutekom , T. Van Der Poll ,Serum concentrations of cytokines in patients with active tuberculosis TB and after treatment, Clin Exp Immunol. 115 1999 110–3.

11 Xie. YongPing; Li. GuoMei; Chen. GuangXing ,Detection serum IL–2 level in patients with active tuberculosis before and after therapy, Modern Preventive Medicine. 36 2009 4382–4383

12 M.K. Katti. Assessment of serum IL–1, IL–2 and IFN–γ levels in untreated pulmonary tuberculosis

patients: role in pathogenesis, Arch Med Res. 42 2011 199–201.

13 S.S. Caner, D. Köksal, S. Ozkara, M. Berkoğlu, S. Aksaray, D. Tarhan, The relation of serum interleukin–2 and C–reactive protein levels with clinical and radiological findings in patients with pulmonary tuberculosis, Tuberk Toraks. 55 2007 238–45.

14 S. Tang , H. Xiao , Y. Fan , F. Wu , Z. Zhang , H. Li , Y. Yang , Changes of proinflammatory cytokines and their receptors in serum from patients with pulmonary tuberculosis, Zhonghua Jie He He Hu Xi Za Zhi. 25 2002 325–9.

15 T.C. Tsao , J. Hong , C. Huang , P. Yang , S.K. Liao , K.S. Chang , Increased TNF–alpha, IL–1 beta and IL–6 levels in the bronchoalveolar lavage fluid with the upregulation of their mRNA in macrophages lavaged from patients with active pulmonary tuberculosis, Tuber Lung Dis. 79 1999 279–85.

16 F. Poveda , J. Camacho , F. Arnalich , R. Codoceo , A. del Arco , P. Martínez–Hernández, Circulating cytokine concentrations in tuberculosis and other chronic bacterial infections, Infection. 27 1999 272–4.

17 F. Deveci , H.H. Akbulut , T. Turgut , M.H. Muz , Changes in serum cytokine levels in active tuberculosis with treatment, Mediators Inflamm. 2005 2005 256–62.

18 R. Rajalingam , N.K. Mehra , J.N. Pande , R.C. Jain , R. Singla , Correlation of serum interleukin–2 receptor alpha levels with clinical manifestations in pulmonary tuberculosis, Tuber Lung Dis. 77 1996 374–9.

19 V. Gupta , A. Jaiswal , D. Behera , H.K.Prasad ,Disparity in circulating peripheral blood dendritic cell subsets and cytokine profile of pulmonary tuberculosis patients compared with healthy family contacts, Hum Immunol. 71 2010 682–91. Epub 2010 Apr 23.

20 F. Ameglio , M. Casarini , E. Capoluongo , P. Mattia , G. Puglisi , S. Giosuè ,Post–treatment changes of six cytokines in active pulmonary tuberculosis: differences between patients with stable or increased fibrosis, Int J Tuberc Lung Dis. 9 2005 98–104.

21 W. Matsuyama , T. Hashiguchi , K. Matsumuro , F. Iwami , Y. Hirotsu , M .Kawabata , K. Arimura , M. Osame , Increased serum level of vascular endothelial growth factor in pulmonary tuberculosis, Am J Respir Crit Care Med. 162 2000 1120–2.

22 C. Marie , M.R. Losser , C. Fitting , N. Kermarrec , D. Payen , J.M. Cavaillon ,Cytokines and soluble cytokine receptors in pleural effusions from septic and nonseptic patients, Am J Respir Crit Care Med. 156 1997 1515–22.

23 L.Kart , H. Buyukoglan , I.O. Tekin , R. Altin , Z. Senturk , I. Gulmez , R. Demir , M. Ozesmi, Correlation of serum tumor necrosis factor–alpha, interleukin–4 and soluble interleukin–2 receptor levels with radiologic and clinical manifestations in active pulmonary tuberculosis, Mediators Inflamm. 12 2003 9–14.

24 D.R. Andrade Júnior , S.A. Santos , I. Castro , D.R. Andrade , Correlation between serum tumor necrosis factor alpha levels and clinical severity of tuberculosis, Braz J Infect Dis. 122008 226–33.

25 Z.T. Handzel , V. Barak , Y. Altman , H. Bibi , M . Lidgi , M. Iancovici–Kidon , D. Yassky , M .Raz ,Increased Th1 and Th2 type cytokine production in patients with active tuberculosis, Isr Med Assoc J. 9 2007 479–83.

26 Z. Hasan , B. Jamil , J. Khan , R. Ali , M.A. Khan , N. Nasir , M.S. Yusuf , S. Jamil , M. Irfan , R. Hussain .Relationship between circulating levels of IFN–gamma, IL–10, CXCL9 and CCL2 in pulmonary and extrapulmonary tuberculosis is dependent on disease severity, Scand J Immunol. 69 2009 259–67.

27 E. Aktas , F. Ciftci , S. Bilgic , O. Sezer , E. Bozkanat , O. Deniz , U. Citici , G. Deniz , Peripheral immune response in pulmonary tuberculosis, Scand J Immunol. 70 2009 300–8.

28 M. Morosini , F. Meloni , A. Marone Bianco , E . Paschetto , M. Uccelli , E. Pozzi , A. Fietta , The assessment of IFN–gamma and its regulatory cytokines in the plasma and bronchoalveolar lavage fluid of patients with active pulmonary tuberculosis, Int J Tuberc Lung Dis.7 2003 994–1000.

29 G. Fiorenza , L. Rateni , M.A. Farroni , C. Bogué , D.G. Dlugovitzky , TNF–alpha, TGF–beta and NO relationship in sera from tuberculosis TB patients of different severity, Immunol Lett. 98 2005 45–8.

30 M. Akgun , L. Saglam , H. Kaynar , A.K. Yildirim , A. Mirici , M. Gorguner , M. Meral , K .Ozden , Serum IL–18 levels in tuberculosis: comparison with pneumonia, lung cancer and healthy controls, Respirology. 10 2005 295–9.

31 L. Wang , Y. Cai , Q. Cheng , Y. Hu , H. Xiao , Imbalance of Th1/Th2 cytokines in patients with pulmonary tuberculosis, Zhonghua Jie He He Hu Xi Za Zhi. 25 2002 535–7.

32 A.C. Leandro , M.A. Rocha , C.S. Cardoso , M.G. Bonecini–Almeida . Genetic polymorphisms in vitamin D receptor, vitamin D–binding protein, Toll–like receptor 2, nitric oxide synthase 2, and interferon–gamma genes and its association with susceptibility to tuberculosis, Braz J Med Biol Res. 42 2009 312–22.
