# Supplementary material for: Establishment of Elevated Serum Levels of IL-10, IL-8 and TNF-β as Potential Peripheral Blood Biomarkers in Tubercular Lymphadenitis: A Prospective Observational Cohort Study
Source: PLoS One. 2016 Jan 19;11(1):e0145576. doi: 10.1371/journal.pone.0145576 (PMC4718686; doi:10.1371/journal.pone.0145576)
Supplement: S3 Appendix — (DOC) [file pone.0145576.s003.doc]

**S3 Appendix**

**Cytokine profile of LNTB as observed in the study.**

The pro-inflammatory response was characterized by IL-18 and TNF-β, which seems to be the profile for tuberculosis in north Indians in wake of a protean IFN-γ and TNF-α response. The main inducer of IFN-γ, IL-12 has been hailed to be efficacious at low levels [1]. In our cases the mean serum IL-12 was found to be very high. It is possible that here IL-12 might have played a dual role: of initially inducing IFN-γ but over induction due to high levels of IL-12 may itself be hampering its production. Elevated serum IL-8 could be indicative of promoting the enhanced chemotactic response which is required in small areas like lymph nodes. But the major difference between PTB and LNTB was the elevated serum IL-10 levels, which according to their reported dual role [2] might be protecting against overtly high pro-inflammatory response and also aid in the persistence of the bacteria by interfering with immunity. It becomes pertinent to point out that apart from presenting the evidence of these putative biomarkers, ours is a first study to address the role of TNF-β in both PTB and LNTB. Our study shows that though TNF-β which has been shown not to function independently of TNF-α [3] can have clinical implications in the outcome of disease and should not be neglected while investigating tuberculosis biomarkers.

References:

1 Leandro AC, Rocha MA, Cardoso CS, Bonecini-Almeida MG. Genetic polymorphisms in vitamin D receptor, vitamin D-binding protein, Toll-like receptor 2, nitric oxide synthase 2, and interferon-gamma genes and its association with susceptibility to tuberculosis. *Braz J Med Biol Res* 2009; 424:312–22.

2 Mege JL , Meghari S, Honstettre A , Capo C , Raoult D . The two faces of interleukin 10 in human infectious diseases. *Lancet Infect Dis* 2006; 557–69.

3 Allie N, Keeton R, Court N, Abel B, Fick L, Vasseur V et al. Limited role for lymphotoxin α in the host immune response to Mycobacterium tuberculosis. *J Immunol* 2010; 185:4292–301.
