# Supplementary material for: Establishment of Elevated Serum Levels of IL-10, IL-8 and TNF-β as Potential Peripheral Blood Biomarkers in Tubercular Lymphadenitis: A Prospective Observational Cohort Study
Source: PLoS One. 2016 Jan 19;11(1):e0145576. doi: 10.1371/journal.pone.0145576 (PMC4718686; doi:10.1371/journal.pone.0145576)
Supplement: S1 Table — (DOCX) [file pone.0145576.s007.docx]

**S1 Table. Showing the results of the test for normality of the data**

| **Tests of Normality** | | | | | | |
| --- | --- | --- | --- | --- | --- | --- |
|  | Kolmogorov-Smirnov^a^ | | | Shapiro-Wilk | | |
|  | Statistic | df | Sig. | Statistic | df | Sig. |
| TNFA | .229 | 215 | .000 | .523 | 215 | .000 |
| IFNG | .171 | 215 | .000 | .818 | 215 | .000 |
| IL6 | .421 | 215 | .000 | .181 | 215 | .000 |
| IL10 | .204 | 215 | .000 | .805 | 215 | .000 |
| IL4 | .182 | 215 | .000 | .679 | 215 | .000 |
| IL18 | .300 | 215 | .000 | .612 | 215 | .000 |
| IL8 | .360 | 215 | .000 | .405 | 215 | .000 |
| IL1RA | .240 | 215 | .000 | .610 | 215 | .000 |
| TNFB | .248 | 215 | .000 | .671 | 215 | .000 |
| IL1B | .397 | 215 | .000 | .147 | 215 | .000 |
| IL2 | .410 | 215 | .000 | .171 | 215 | .000 |
| IL12 | .361 | 215 | .000 | .227 | 215 | .000 |
| a. Lilliefors Significance Correction | | | | | | |
