# Supplementary material for: Establishment of Elevated Serum Levels of IL-10, IL-8 and TNF-β as Potential Peripheral Blood Biomarkers in Tubercular Lymphadenitis: A Prospective Observational Cohort Study
Source: PLoS One. 2016 Jan 19;11(1):e0145576. doi: 10.1371/journal.pone.0145576 (PMC4718686; doi:10.1371/journal.pone.0145576)
Supplement: S2 Table — (DOCX) [file pone.0145576.s008.docx]

**S2 Table. Confusion matrix of testing set for classification of the LAP classes using the decision tree model**

| **Class label** | **Cancerous LAP^#^** | **LNTB** | **Other LAP** |
| --- | --- | --- | --- |
| **Cancerous LAP^*^** | 13 | 5 | 0 |
| **LNTB** | 2 | 34 | 0 |
| **Other LAP** | 0 | 4 | 3 |

^#^Column names represent the true class labels; ^*^row names are the predicted class labels from the model.
