# Supplementary material for: Establishment of Elevated Serum Levels of IL-10, IL-8 and TNF-β as Potential Peripheral Blood Biomarkers in Tubercular Lymphadenitis: A Prospective Observational Cohort Study
Source: PLoS One. 2016 Jan 19;11(1):e0145576. doi: 10.1371/journal.pone.0145576 (PMC4718686; doi:10.1371/journal.pone.0145576)
Supplement: S3 Table — (DOCX) [file pone.0145576.s009.docx]

**S3 Table. Class wise accuracy for the decision tree model**

| **Class label** | **Sensitivity** | **Specificity** | **PPV** | **NPV** |
| --- | --- | --- | --- | --- |
| **Cancerous LAP^*^** | 88.5 | 100 | 100 | 95 |
| **LNTB** | 100 | 83.6 | 87.5 | 100 |
| **Other LAP** | 75 | 100 | 100 | 95 |

The data expressed as percentage. PPV: Positive predictive value; NPV: Negative Predictive value.
