# Supplementary material for: Establishment of Elevated Serum Levels of IL-10, IL-8 and TNF-β as Potential Peripheral Blood Biomarkers in Tubercular Lymphadenitis: A Prospective Observational Cohort Study
Source: PLoS One. 2016 Jan 19;11(1):e0145576. doi: 10.1371/journal.pone.0145576 (PMC4718686; doi:10.1371/journal.pone.0145576)
Supplement: S4 Table — (DOCX) [file pone.0145576.s010.docx]

**S4 Table. Total sample size in each of the three classes used for multinomial modelling**

| **Class Label** | **Sample Size (n)** |
| --- | --- |
| Lymph node TB (LNTB) | 63 |
| Cancerous LAP | 35 |
| Other LAP | 20 |
