# Supplementary material for: Establishment of Elevated Serum Levels of IL-10, IL-8 and TNF-β as Potential Peripheral Blood Biomarkers in Tubercular Lymphadenitis: A Prospective Observational Cohort Study
Source: PLoS One. 2016 Jan 19;11(1):e0145576. doi: 10.1371/journal.pone.0145576 (PMC4718686; doi:10.1371/journal.pone.0145576)
Supplement: S5 Table — (DOCX) [file pone.0145576.s011.docx]

**S5 Table. Sample size for modelling using balanced sampling**

| **Class label** | **Cancerous LAP** | **LNTB** | **Other LAP** |
| --- | --- | --- | --- |
| Training set (n) | 20 | 20 | 17 |
| Test set (n) | 15 | 43 | 3 |
