# Supplementary material for: Establishment of Elevated Serum Levels of IL-10, IL-8 and TNF-β as Potential Peripheral Blood Biomarkers in Tubercular Lymphadenitis: A Prospective Observational Cohort Study
Source: PLoS One. 2016 Jan 19;11(1):e0145576. doi: 10.1371/journal.pone.0145576 (PMC4718686; doi:10.1371/journal.pone.0145576)
Supplement: S6 Table — (DOCX) [file pone.0145576.s012.docx]

**S6 Table. Sample size for modelling using proportionate sampling**

| **Class label** | **Cancerous LAP** | **LNTB** | **Other LAP** |
| --- | --- | --- | --- |
| Training set (n) | 28 | 50 | 16 |
| Test set (n) | 7 | 13 | 4 |
