# Supplementary material for: Establishment of Elevated Serum Levels of IL-10, IL-8 and TNF-β as Potential Peripheral Blood Biomarkers in Tubercular Lymphadenitis: A Prospective Observational Cohort Study
Source: PLoS One. 2016 Jan 19;11(1):e0145576. doi: 10.1371/journal.pone.0145576 (PMC4718686; doi:10.1371/journal.pone.0145576)
Supplement: S7 Table — (DOCX) [file pone.0145576.s013.docx]

**S7 Table: Confusion matrix of testing set for classification of the LAP classes using the model built with balanced training set**

| **Class label** | **Cancerous LAP^#^** | **LNTB** | **Other LAP** |
| --- | --- | --- | --- |
| **Cancerous LAP^*^** | 13 | 5 | 0 |
| **LNTB** | 2 | 34 | 0 |
| **Other LAP** | 0 | 4 | 3 |

^#^Column names represent the true class labels; ^*^row names are the predicted class labels from the model. It may be seen only two cancerous LAP samples were incorrectly predicted as LNPTB and that none of the other lymphadenopathy samples got incorrectly predicted.
