# Supplementary material for: Establishment of Elevated Serum Levels of IL-10, IL-8 and TNF-β as Potential Peripheral Blood Biomarkers in Tubercular Lymphadenitis: A Prospective Observational Cohort Study
Source: PLoS One. 2016 Jan 19;11(1):e0145576. doi: 10.1371/journal.pone.0145576 (PMC4718686; doi:10.1371/journal.pone.0145576)
Supplement: S8 Table — (DOCX) [file pone.0145576.s014.docx]

**S8 Table: Confusion matrix of testing set for classification of the LAP classes using the model built with proportionate training set**

| **Class label** | **Cancerous LAP^#^** | **LNTB** | **Other LAP** |
| --- | --- | --- | --- |
| **Cancerous LAP^*^** | 7 | 1 | 0 |
| **LNTB** | 0 | 12 | 0 |
| **Other LAP** | 0 | 0 | 4 |

^#^Column names represent the true class labels; ^*^row names are the predicted class labels from the model. It shows 95.83% overall accuracy. It may be seen only one LNPTB sample was incorrectly predicted as cancerous, while all other samples were correctly predicted.
