# Supplementary material for: Establishment of Elevated Serum Levels of IL-10, IL-8 and TNF-β as Potential Peripheral Blood Biomarkers in Tubercular Lymphadenitis: A Prospective Observational Cohort Study
Source: PLoS One. 2016 Jan 19;11(1):e0145576. doi: 10.1371/journal.pone.0145576 (PMC4718686; doi:10.1371/journal.pone.0145576)
Supplement: S9 Table — (DOCX) [file pone.0145576.s015.docx]

**S9 Table: Decision tree model original and predicted class**

| **Serial number** | **Sample ID** | **Original Class** | **Mean serum cytokine levels (pg/ml)** | | | **Predicted Class** |
| --- | --- | --- | --- | --- | --- | --- |
|  |  |  | **IL- 10** | **IL- 8** | **TNF-b** |  |
| 1 | Sample33 | Cancerous LAP | 86.89522 | 853.278 | 712.1242 | LNPTB |
| 2 | Sample3 | Cancerous LAP | 83.5266 | 6172.89 | 8431.522 | Cancerous LAP |
| 3 | Sample17 | Cancerous LAP | 78.84025 | 1691.109 | 9112.191 | Cancerous LAP |
| 4 | Sample1 | Cancerous LAP | 63.85016 | 2370.604 | 9212.877 | Cancerous LAP |
| 5 | Sample29 | Cancerous LAP | 86.19463 | 499.8885 | 2805.837 | Cancerous LAP |
| 6 | Sample31 | Cancerous LAP | 59.46895 | 396.752 | 3569.437 | Cancerous LAP |
| 7 | Sample11 | Cancerous LAP | 72.58642 | 2279.055 | 8247.516 | Cancerous LAP |
| 8 | Sample12 | Cancerous LAP | 86.89522 | 5445.735 | 8565.369 | Cancerous LAP |
| 9 | Sample16 | Cancerous LAP | 93.76375 | 1442.342 | 8730.51 | Cancerous LAP |
| 10 | Sample4 | Cancerous LAP | 86.19463 | 3747.646 | 8904.982 | Cancerous LAP |
| 11 | Sample30 | Cancerous LAP | 61.02178 | 523.3885 | 257.6818 | LNPTB |
| 12 | Sample2 | Cancerous LAP | 72.80679 | 1845.833 | 8247.516 | Cancerous LAP |
| 13 | Sample34 | Cancerous LAP | 69.85044 | 370 | 8685.302 | Cancerous LAP |
| 14 | Sample27 | Cancerous LAP | 72.80679 | 2459.824 | 712.1242 | LNPTB |
| 15 | Sample20 | Cancerous LAP | 100.4355 | 499.8885 | 2942.239 | Cancerous LAP |
| 16 | Sample24 | Cancerous LAP | 25.90158 | 1254.967 | 2938.055 | Cancerous LAP |
| 17 | Sample13 | Cancerous LAP | 69.85044 | 2294.239 | 8520.627 | Cancerous LAP |
| 18 | Sample32 | Cancerous LAP | 72.58642 | 872.935 | 8394.546 | Cancerous LAP |
| 19 | Sample21 | Cancerous LAP | 100.0676 | 523.3885 | 2842.69 | Cancerous LAP |
| 20 | Sample26 | Cancerous LAP | 67.36441 | 1105.282 | 8394.546 | Cancerous LAP |
| 21 | Sample94 | LNTB | 0 | 33.32607 | 5574.147 | LNPTB |
| 22 | Sample40 | LNTB | 41.60491 | 166.5114 | 9740.985 | LNPTB |
| 23 | Sample66 | LNTB | 37.9448 | 88.61788 | 29.20166 | LNPTB |
| 24 | Sample36 | LNTB | 45.69115 | 142.5846 | 1887.25 | LNPTB |
| 25 | Sample90 | LNTB | 22.44678 | 0.238107 | 16198.49 | LNPTB |
| 26 | Sample91 | LNTB | 15.23318 | 173.6058 | 6649.361 | LNPTB |
| 27 | Sample56 | LNTB | 79.53 | 1662.134 | 141.6177 | LNPTB |
| 28 | Sample59 | LNTB | 43.32204 | 162.0395 | 193.7933 | LNPTB |
| 29 | Sample67 | LNTB | 31.1105 | 45.54904 | 56.07152 | LNPTB |
| 30 | Sample43 | LNTB | 37.6479 | 145.236 | 2085.354 | LNPTB |
| 31 | Sample57 | LNTB | 76.81692 | 1036.376 | 272.996 | LNPTB |
| 32 | Sample39 | LNTB | 42.63438 | 155.5422 | 1859.823 | LNPTB |
| 33 | Sample41 | LNTB | 110.5339 | 176.0174 | 1504.612 | LNPTB |
| 34 | Sample71 | LNTB | 96.94652 | 80.36884 | 1635.528 | LNPTB |
| 35 | Sample82 | LNTB | 41.9583 | 0.763701 | 999.5576 | LNPTB |
| 36 | Sample38 | LNTB | 32.07294 | 143.7949 | 1480.361 | LNPTB |
| 37 | Sample98 | LNTB | 0 | 0 | 0 | LNPTB |
| 38 | Sample83 | LNTB | 15.64126 | 13.99534 | 1184.74 | LNPTB |
| 39 | Sample86 | LNTB | 16.05152 | 22.09881 | 2553.34 | LNPTB |
| 40 | Sample45 | LNTB | 47.98806 | 145.4591 | 1839.395 | LNPTB |
| 41 | Sample117 | Other LAP | 13.88319 | 26.41242 | 252.1163 | Other LAP |
| 42 | Sample100 | Other LAP | 20.82479 | 34.65883 | 252.1163 | Other LAP |
| 43 | Sample107 | Other LAP | 18.58796 | 8.105135 | 219.0726 | LNPTB |
| 44 | Sample99 | Other LAP | 21.57798 | 33.71155 | 414.1695 | Other LAP |
| 45 | Sample113 | Other LAP | 18.11972 | 6.089692 | 252.1163 | Other LAP |
| 46 | Sample114 | Other LAP | 10.38152 | 0.513615 | 252.1163 | Other LAP |
| 47 | Sample103 | Other LAP | 0 | 0.364028 | 244.4349 | Other LAP |
| 48 | Sample104 | Other LAP | 0 | 1.18318 | 308.2774 | Other LAP |
| 49 | Sample105 | Other LAP | 15.57228 | 0.813495 | 506.0007 | Other LAP |
| 50 | Sample118 | Other LAP | 10.38152 | 20.18897 | 219.0726 | LNPTB |
| 51 | Sample112 | Other LAP | 18.08864 | 20.18897 | 414.1695 | Other LAP |
| 52 | Sample115 | Other LAP | 11.40245 | 0.095362 | 219.0726 | LNPTB |
| 53 | Sample116 | Other LAP | 12.39197 | 6.861552 | 436.1957 | Other LAP |
| 54 | Sample108 | Other LAP | 20.82479 | 20.27136 | 436.1957 | Other LAP |
| 55 | Sample111 | Other LAP | 21.38064 | 10.48043 | 506.0007 | Other LAP |
| 56 | Sample106 | Other LAP | 17.10368 | 208.7286 | 291.265 | LNPTB |
| 57 | Sample101 | Other LAP | 18.58796 | 8.105135 | 219.0726 | LNPTB |
| 58 | Sample5 | Cancerous LAP | 61.02178 | 8300.605 | 9127.64 | Cancerous LAP |
| 59 | Sample6 | Cancerous LAP | 59.46895 | 872.935 | 9012.103 | Cancerous LAP |
| 60 | Sample7 | Cancerous LAP | 58.74887 | 853.278 | 8753.16 | Cancerous LAP |
| 61 | Sample8 | Cancerous LAP | 66.82794 | 1524.616 | 8357.657 | Cancerous LAP |
| 62 | Sample9 | Cancerous LAP | 53.19363 | 3094.471 | 8692.825 | Cancerous LAP |
| 63 | Sample10 | Cancerous LAP | 62.90263 | 4552.529 | 9212.877 | Cancerous LAP |
| 64 | Sample14 | Cancerous LAP | 69.95922 | 1113.221 | 9050.525 | Cancerous LAP |
| 65 | Sample15 | Cancerous LAP | 88.06703 | 2526.917 | 8775.845 | Cancerous LAP |
| 66 | Sample18 | Cancerous LAP | 87.48047 | 1801.141 | 9765.356 | Cancerous LAP |
| 67 | Sample19 | Cancerous LAP | 108.892 | 5585.085 | 2925.519 | Cancerous LAP |
| 68 | Sample22 | Cancerous LAP | 48.69035 | 396.752 | 8685.302 | Cancerous LAP |
| 69 | Sample23 | Cancerous LAP | 63.00769 | 1525.123 | 9392.487 | Cancerous LAP |
| 70 | Sample25 | Cancerous LAP | 210.5839 | 1165.887 | 3569.437 | Cancerous LAP |
| 71 | Sample28 | Cancerous LAP | 83.5266 | 0.153053 | 3181.756 | LNPTB |
| 72 | Sample35 | Cancerous LAP | 69.95922 | 800 | 9392.487 | Cancerous LAP |
| 73 | Sample37 | LNTB | 37.03921 | 185.8513 | 5026.59 | LNPTB |
| 74 | Sample42 | LNTB | 54.40803 | 157.259 | 1420.67 | LNPTB |
| 75 | Sample44 | LNTB | 39.80413 | 144.5949 | 1752.282 | LNPTB |
| 76 | Sample46 | LNTB | 45.60981 | 164.6581 | 1686.828 | LNPTB |
| 77 | Sample47 | LNTB | 40.74037 | 149.4733 | 1654.605 | LNPTB |
| 78 | Sample48 | LNTB | 42.63438 | 146.0826 | 1887.25 | LNPTB |
| 79 | Sample49 | LNTB | 54.40803 | 144.5949 | 5026.59 | LNPTB |
| 80 | Sample50 | LNTB | 47.98806 | 145.4591 | 1480.361 | LNPTB |
| 81 | Sample51 | LNTB | 47.48852 | 1052.992 | 79.98458 | LNPTB |
| 82 | Sample52 | LNTB | 54.67444 | 294.1693 | 76.10698 | LNPTB |
| 83 | Sample53 | LNTB | 64.18724 | 218.7556 | 79.98458 | LNPTB |
| 84 | Sample54 | LNTB | 67.2822 | 275.0055 | 169.6973 | LNPTB |
| 85 | Sample55 | LNTB | 63.67556 | 584.9568 | 47.8909 | LNPTB |
| 86 | Sample58 | LNTB | 50.80514 | 1086.991 | 296.6822 | LNPTB |
| 87 | Sample60 | LNTB | 46.0873 | 387.5889 | 88.25398 | LNPTB |
| 88 | Sample61 | LNTB | 55.65462 | 484.5056 | 38.59788 | LNPTB |
| 89 | Sample62 | LNTB | 101.0159 | 517.4112 | 21.84858 | LNPTB |
| 90 | Sample63 | LNTB | 132.0791 | 339.2196 | 335.5298 | LNPTB |
| 91 | Sample64 | LNTB | 98.10418 | 69.6688 | 250.9594 | LNPTB |
| 92 | Sample65 | LNTB | 107.5082 | 33.63588 | 169.6973 | LNPTB |
| 93 | Sample68 | LNTB | 36.19934 | 296.6532 | 62.17152 | LNPTB |
| 94 | Sample69 | LNTB | 42.41148 | 175.1066 | 53.2203 | LNPTB |
| 95 | Sample70 | LNTB | 12.31385 | 9.779044 | 65.43034 | LNPTB |
| 96 | Sample72 | LNTB | 135.2524 | 6.594452 | 2935.632 | LNPTB |
| 97 | Sample73 | LNTB | 115.9394 | 1.536687 | 6000 | LNPTB |
| 98 | Sample74 | LNTB | 42.41148 | 0.797779 | 1089.058 | LNPTB |
| 99 | Sample75 | LNTB | 29.46512 | 23.38238 | 1089.058 | LNPTB |
| 100 | Sample76 | LNTB | 27.44564 | 238.1684 | 2739.249 | LNPTB |
| 101 | Sample77 | LNTB | 41.05628 | 17.77484 | 6000 | LNPTB |
| 102 | Sample78 | LNTB | 51.28424 | 1.023751 | 1766.881 | LNPTB |
| 103 | Sample79 | LNTB | 65.21414 | 1.690068 | 1184.74 | LNPTB |
| 104 | Sample80 | LNTB | 95.79284 | 1.89746 | 3142.913 | LNPTB |
| 105 | Sample81 | LNTB | 94.06996 | 0.110043 | 10326.47 | LNPTB |
| 106 | Sample84 | LNTB | 16.05152 | 104.8071 | 999.5576 | LNPTB |
| 107 | Sample85 | LNTB | 16.87844 | 87.8358 | 1184.74 | LNPTB |
| 108 | Sample87 | LNTB | 15.23318 | 9.042932 | 3591.93 | LNPTB |
| 109 | Sample88 | LNTB | 15.23318 | 48.54948 | 1635.528 | LNPTB |
| 110 | Sample89 | LNTB | 20.69946 | 8.780788 | 2553.34 | LNPTB |
| 111 | Sample92 | LNTB | 14.82732 | 52.68604 | 1511.975 | LNPTB |
| 112 | Sample93 | LNTB | 0 | 347.8252 | 1766.881 | LNPTB |
| 113 | Sample95 | LNTB | 0 | 50.34816 | 2739.249 | LNPTB |
| 114 | Sample96 | LNTB | 0 | 0 | 0 | LNPTB |
| 115 | Sample97 | LNTB | 0 | 0 | 0 | LNPTB |
| 116 | Sample102 | Other LAP | 18.71348 | 20.27136 | 436.1957 | Other LAP |
| 117 | Sample109 | Other LAP | 15.48368 | 0.364028 | 244.4349 | Other LAP |
| 118 | Sample110 | Other LAP | 18.55661 | 5.678516 | 308.2774 | Other LAP |
