# Supplementary material for: Establishment of Elevated Serum Levels of IL-10, IL-8 and TNF-β as Potential Peripheral Blood Biomarkers in Tubercular Lymphadenitis: A Prospective Observational Cohort Study
Source: PLoS One. 2016 Jan 19;11(1):e0145576. doi: 10.1371/journal.pone.0145576 (PMC4718686; doi:10.1371/journal.pone.0145576)
Supplement: S10 Table — (DOCX) [file pone.0145576.s016.docx]

**S10 Table. Concordance of the prediction between the decision tree and the multiple logistic regression models**

| **Serial number** | **Original Class** | **Predicted Class** | |
| --- | --- | --- | --- |
|  |  | **Decision Tree** | **Logistic regression** |
| 1 | Cancerous LAP | Cancerous LAP | Cancerous LAP |
| 2 | Cancerous LAP | Cancerous LAP | Cancerous LAP |
| 3 | Cancerous LAP | Cancerous LAP | Cancerous LAP |
| 4 | Cancerous LAP | Cancerous LAP | Cancerous LAP |
| 5 | Cancerous LAP | Cancerous LAP | Cancerous LAP |
| 6 | Cancerous LAP | Cancerous LAP | Cancerous LAP |
| 7 | Cancerous LAP | Cancerous LAP | Cancerous LAP |
| 8 | Cancerous LAP | Cancerous LAP | Cancerous LAP |
| 9 | Cancerous LAP | Cancerous LAP | Cancerous LAP |
| 10 | Cancerous LAP | Cancerous LAP | Cancerous LAP |
| 11 | Cancerous LAP | Cancerous LAP | Cancerous LAP |
| 12 | Cancerous LAP | Cancerous LAP | Cancerous LAP |
| 13 | Cancerous LAP | Cancerous LAP | Cancerous LAP |
| 14 | Cancerous LAP | Cancerous LAP | Cancerous LAP |
| 15 | Cancerous LAP | Cancerous LAP | Cancerous LAP |
| 16 | Cancerous LAP | Cancerous LAP | Cancerous LAP |
| 17 | Cancerous LAP | Cancerous LAP | Cancerous LAP |
| 18 | Cancerous LAP | Cancerous LAP | Cancerous LAP |
| 19 | Cancerous LAP | Cancerous LAP | Cancerous LAP |
| 20 | Cancerous LAP | Cancerous LAP | LNTB |
| 21 | Cancerous LAP | Cancerous LAP | LNTB |
| 22 | Cancerous LAP | Cancerous LAP | LNTB |
| 23 | Cancerous LAP | Cancerous LAP | Cancerous LAP |
| 24 | Cancerous LAP | Cancerous LAP | Cancerous LAP |
| 25 | Cancerous LAP | Cancerous LAP | Cancerous LAP |
| 26 | Cancerous LAP | Cancerous LAP | Cancerous LAP |
| 27 | Cancerous LAP | LNTB | Cancerous LAP |
| 28 | Cancerous LAP | LNTB | LNTB |
| 29 | Cancerous LAP | Cancerous LAP | LNTB |
| 30 | Cancerous LAP | LNTB | LNTB |
| 31 | Cancerous LAP | Cancerous LAP | LNTB |
| 32 | Cancerous LAP | Cancerous LAP | Cancerous LAP |
| 33 | Cancerous LAP | LNTB | LNTB |
| 34 | Cancerous LAP | Cancerous LAP | Cancerous LAP |
| 35 | Cancerous LAP | Cancerous LAP | Cancerous LAP |
| 36 | LNTB | LNTB | LNTB |
| 37 | LNTB | LNTB | LNTB |
| 38 | LNTB | LNTB | LNTB |
| 39 | LNTB | LNTB | LNTB |
| 40 | LNTB | LNTB | LNTB |
| 41 | LNTB | LNTB | LNTB |
| 42 | LNTB | LNTB | LNTB |
| 43 | LNTB | LNTB | LNTB |
| 44 | LNTB | LNTB | LNTB |
| 45 | LNTB | LNTB | LNTB |
| 46 | LNTB | LNTB | LNTB |
| 47 | LNTB | LNTB | LNTB |
| 48 | LNTB | LNTB | LNTB |
| 49 | LNTB | LNTB | LNTB |
| 50 | LNTB | LNTB | LNTB |
| 51 | LNTB | LNTB | LNTB |
| 52 | LNTB | LNTB | LNTB |
| 53 | LNTB | LNTB | LNTB |
| 54 | LNTB | LNTB | LNTB |
| 55 | LNTB | LNTB | LNTB |
| 56 | LNTB | LNTB | Cancerous LAP |
| 57 | LNTB | LNTB | Cancerous LAP |
| 58 | LNTB | LNTB | LNTB |
| 59 | LNTB | LNTB | LNTB |
| 60 | LNTB | LNTB | LNTB |
| 61 | LNTB | LNTB | LNTB |
| 62 | LNTB | LNTB | LNTB |
| 63 | LNTB | LNTB | LNTB |
| 64 | LNTB | LNTB | LNTB |
| 65 | LNTB | LNTB | LNTB |
| 66 | LNTB | LNTB | LNTB |
| 67 | LNTB | LNTB | Other LAP |
| 68 | LNTB | LNTB | LNTB |
| 69 | LNTB | LNTB | LNTB |
| 70 | LNTB | LNTB | Other LAP |
| 71 | LNTB | LNTB | LNTB |
| 72 | LNTB | LNTB | LNTB |
| 73 | LNTB | LNTB | LNTB |
| 74 | LNTB | LNTB | LNTB |
| 75 | LNTB | LNTB | LNTB |
| 76 | LNTB | LNTB | LNTB |
| 77 | LNTB | LNTB | LNTB |
| 78 | LNTB | LNTB | LNTB |
| 79 | LNTB | LNTB | LNTB |
| 80 | LNTB | LNTB | LNTB |
| 81 | LNTB | LNTB | Cancerous LAP |
| 82 | LNTB | LNTB | LNTB |
| 83 | LNTB | LNTB | LNTB |
| 84 | LNTB | LNTB | LNTB |
| 85 | LNTB | LNTB | LNTB |
| 86 | LNTB | LNTB | LNTB |
| 87 | LNTB | LNTB | LNTB |
| 88 | LNTB | LNTB | LNTB |
| 89 | LNTB | LNTB | LNTB |
| 90 | LNTB | LNTB | LNTB |
| 91 | LNTB | LNTB | LNTB |
| 92 | LNTB | LNTB | LNTB |
| 93 | LNTB | LNTB | LNTB |
| 94 | LNTB | LNTB | LNTB |
| 95 | LNTB | LNTB | LNTB |
| 96 | LNTB | LNTB | Other LAP |
| 97 | LNTB | LNTB | Other LAP |
| 98 | LNTB | LNTB | Other LAP |
| 99 | Other LAP | Other LAP | Other LAP |
| 100 | Other LAP | Other LAP | Other LAP |
| 101 | Other LAP | LNTB | Other LAP |
| 102 | Other LAP | Other LAP | Other LAP |
| 103 | Other LAP | Other LAP | Other LAP |
| 104 | Other LAP | Other LAP | Other LAP |
| 105 | Other LAP | Other LAP | Other LAP |
| 106 | Other LAP | LNTB | LNTB |
| 107 | Other LAP | LNTB | Other LAP |
| 108 | Other LAP | Other LAP | Other LAP |
| 109 | Other LAP | Other LAP | Other LAP |
| 110 | Other LAP | Other LAP | Other LAP |
| 111 | Other LAP | Other LAP | Other LAP |
| 112 | Other LAP | Other LAP | Other LAP |
| 113 | Other LAP | Other LAP | Other LAP |
| 114 | Other LAP | Other LAP | Other LAP |
| 115 | Other LAP | LNTB | Other LAP |
| 116 | Other LAP | Other LAP | Other LAP |
| 117 | Other LAP | Other LAP | Other LAP |
| 118 | Other LAP | LNTB | Other LAP |

Concordance percentage between the two when decision tree is compared to the logistic regression model is: LNTB (87.3), Cancerous LAP (77.14), Other LAP (75%).
